# Whole‐exome sequencing in clear cell sarcoma of soft tissue uncovers novel prognostic categorization and drug targets

**DOI:** 10.1002/ctm2.640

**Published:** 2021-12-19

**Authors:** Jingjing Li, Chao Chen, Wei Liu, Songming Liu, Wanming Hu, Xiaoyan Gao, Geng Liu, Dandan Li, Ya Ding, Xizhi Wen, Xiuqing Zhang, Yong Hou, Xing Zhang, Bo Li, Xiaoshi Zhang, Xi Zhang

**Affiliations:** ^1^ Biotherapy Center Sun Yat‐sen University Cancer Center Sun Yat‐sen University Guangzhou China; ^2^ Beijing Genomics Institute Shenzhen China; ^3^ State Key Laboratory of Oncology in South China Sun Yat‐sen University Guangzhou China; ^4^ Collaborative Innovation Center for Cancer Medicine Guangzhou China; ^5^ China National GeneBank Beijing Genomics Institute Shenzhen China; ^6^ Department of Pathology Sun Yat‐sen University Cancer Center Guangzhou China

Dear Editor,

In this study, we addressed the clinical outcome of clear cell sarcoma (CCS) of soft tissue by conducting the first mutational landscape of CCS. We also revealed the potential of targeted therapies in CCS, suggesting that CCS patients with ataxia‐telangiectasia mutated alterations may be likely to benefit from treatment with an ataxia telangiectasia and Rad3‐related inhibitor.

CCS is a rare soft tissue sarcoma first reported by Enzinger in 1965.^1^ Due to the rarity and the difficulty in the diagnosis of CCS, the genomic characteristics of CCS have not been systemically investigated and the pathogenesis and optimal treatment have not been determined. Therefore, we retrospectively investigated 21 CCS samples from Sun Yat‐sen University Cancer Center and conducted high‐depth whole‐exome sequencing (WES) on these samples (Figure S1 and Tables S1 and S2). We confirmed the pathologic diagnosis by the gene fusion/translocation detection of *EWSR1*.

First, the somatic mutation patterns of CCS were identified. A total of 1949 mutations were detected (Figure 1A and Table S3). The median mutation number was 77, which was comparable with other subtypes of sarcoma in the cancer genome atlas sarcoma (TCGA‐SARC) (Figures S2 and S3). The predominant somatic mutation type was C: G > T: A transitions at CpG dinucleotides (Figure S4), reflecting an age‐related mutation pattern.^2^ We further identified the signatures of mutations, resulting in two stable signatures (Figure 1B, Figures S5 and S6 and Table S4). A total of 66 cancer‐related genes were mutated in these samples, nine of which occurred in at least two samples (Table S5). Interestingly, we found that two tumour samples harboured a hotspot mutation (rs1242535815) in the promoter region of *TERT*, which is a driver gene in many cancers.^3^, ^4^ We further found that four out of 35 CCS cases (11.4%) in the genomics evidence neoplasia information exchange (GENIE) database (version 9.0)^5^ also carried the rs1242535815 mutation (Table S3), suggesting that this mutation is a potential driver of CCS. In Figure S7, the top 25 mutated cancer‐related genes were listed in these two cohorts (*N* = 56), and 16 of them were recurrently mutated genes (Figure 1C).

**FIGURE 1:**
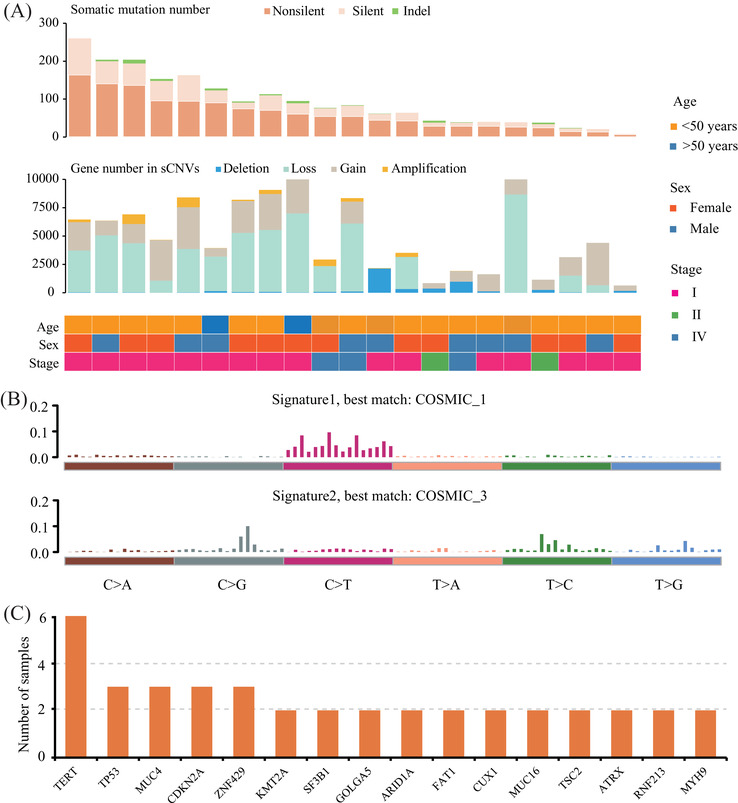
Mutation landscape of clear cell carcoma (CCS). (A) The number of somatic mutations and copy number altered genes for each CCS sample in this study (*N* = 21). Sex, age, and tumor stages are listed at the bottom according to the samples. (B) The signatures are displayed in the 96‐substitution classification, with the X‐axis representing the mutation types and the Y‐axis representing the estimated mutation for each mutation type, identified by Bayesian non‐negative matrix factorization (NMF) algorithm; (C) The recurrent mutated cancer‐related genes in this study (*N* = 21) and in the GENIE cohort (*N* = 26)

Second, widespread somatic copy number variations (CNVs) were detected in 21 CCS tumours (Figure 1A). Frequent arm‐level alterations included copy number gains in 7p (33%, *q* = .00125), 7q (29%, *q* = .00343), 8p (48%, *q* < .0001), and 8q (71%, *q* < .0001) and copy number losses in 16 (33%, *q* = .0008), 19p (33%, *q* = .0067), and 19q (33%, *q* = .0013) (Figure 2A and Table S6). The alterations of chromosomes 7 and 8 were similar to those in melanoma, while the frequent losses of chromosome 16 were similar to those in TCGA‐SARC (Figure S3 and Table S7). Twenty focal copy number amplifications and 18 focal copy number deletions were identified using genomic identification of significant targets in cancer 2.0^6^ (Figure 2B and Tables S8 and S9). Previous studies showed that the deletion of 9p21.3 is negatively correlated with the prognosis of lymphoepithelioma‐like carcinoma.^7^ In this study, the deletion of the 9p21.3 region was observed in 38% of the patients (8/21, Figure 2C), and this deletion was negatively correlated with the relapse‐free survival and overall survival of the CCS patients (Figure 2D,E).

**FIGURE 2:**
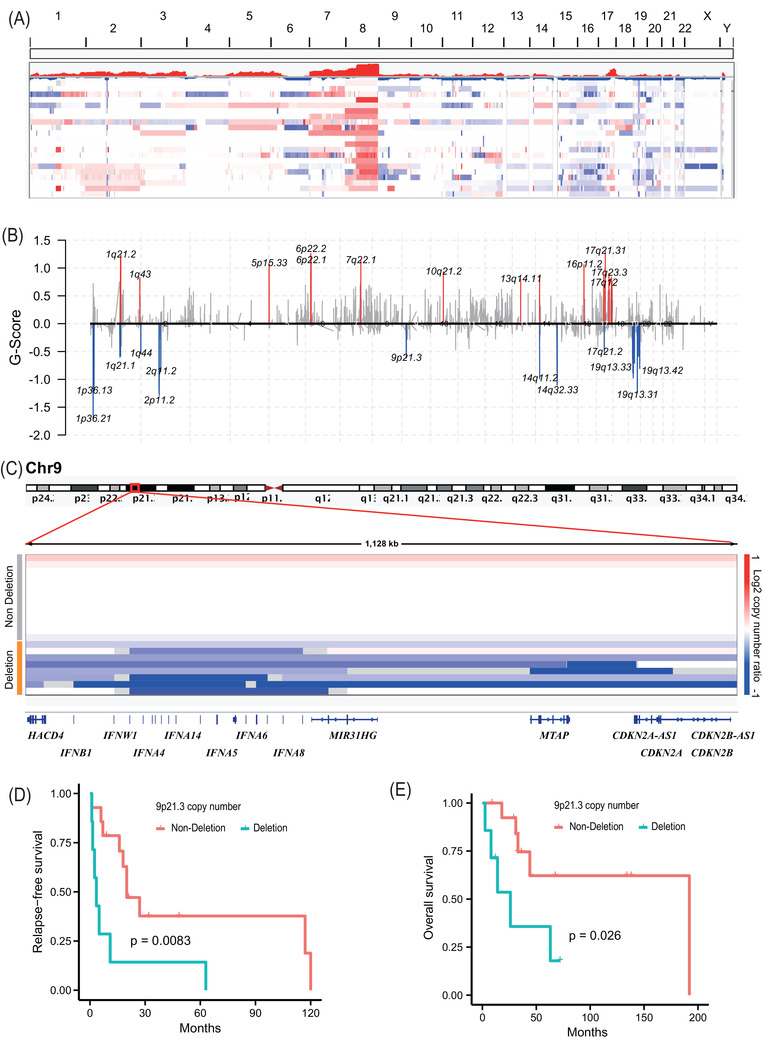
Somatic copy number variations (CNVs) of clear cell carcoma (CCS) in this study. (A) CNV landscape of 21 CCS samples. (B) Focal amplification (red) and deletion (blue) across CCS samples. (C) The deletion (including heterozygous deletion and homozygous deletion) of 9p21.3. Relapse‐free survival (D) and overall survival (E) analysis for CNV status of 9p21.3. Statistical significance was estimated by a two‐sided log‐rank test

Third, according to the gene set enrichment analysis^8^ of the genes with CNV, we found that these genes were involved in several cancer‐related pathways, including the antigen processing and presentation, the Janus kinase/signal transducer and activator of transcription (JAK/STAT) signalling pathway, p53 pathway, and the cell cycle (Figure S8). The p53 pathway was altered primarily by deletion of *TP53*, followed by deletion of *CDKN2A*, *CHEK2* and *ATM* (Figure 3A). The JAK/STAT pathway was frequently dysregulated, mainly due to the deletion of suppressors of cytokine signalling family genes and *PTPRD* (Figure 3B). We found that the change in the cell cycle pathway was related not only to the deletion of *CDKN2A* but also to the amplification of *CDK2*/*4/6* (Figure 3C). To validate our findings, we analysed the somatic CNVs of CCS in the GENIE database (Figure S9). Furthermore, we performed fluorescence in situ hybridization (FISH) tests for *CDK4* and *MDM2* amplification in the two tumour samples available. The results showed that the CNVs detected by FISH were consistent with those detected by WES (Figures S10 and S11). In addition, RNA sequencing was performed on six CCS samples, *HGF* and *MET* were found to be upregulated, and CD8+ cell infiltration was low (Figure S12).

**FIGURE 3:**
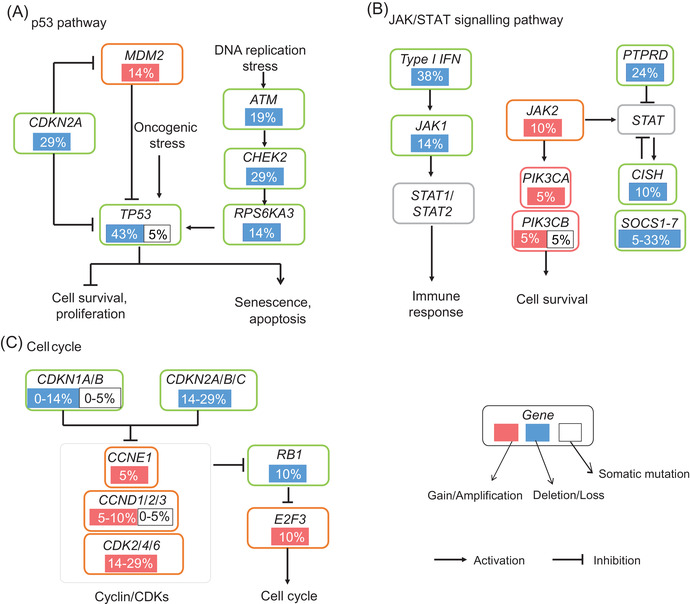
Pathways altered in clear cell carcoma (CCS). Frequent mutations, including somatic mutations, amplifications, and deletions, were enriched in the p53 (A) Janus kinase/signal transducer and activator of transcription (JAK/STAT) (B), and cell cycle (C) pathways. The mutation frequency is expressed as a percentage of the CCS cohort in this study(*N* = 21)

Finally, as the deletion of *ATM* and *CHEK2* occurred with a relatively high frequency in CCS, we wanted to know whether inhibition of other DDR pathways might lead to the lethal synthesis of CCS. We tested two highly selective DDR inhibitors that are either currently used in the clinics (poly ADP‐ribose polymerase (PARP) inhibitor‐olaparib) or clinical trials (ATR inhibitor‐AZD6738).^9^, ^10^ We performed the assays on a CCS cell line (SU‐CCS‐1) that we could find in the American type culture collection. SU‐CCS‐1 cells were isolated from the pleural effusion of a patient with CCS, which showed a heterozygous loss of *ATM* at the CNV level, and a decrease in the protein levels of ATM and CHK2 in the cells (Figure 4A and Figure S13). When we treated the cells with either olaparib or AZD6738, from the cell viability assay, we observed that AZD6738 significantly reduced the viability of SU‐CCS‐1 cells, with an IC50 of 0.5μM. On the other hand, olaparib could only reduce the viability of SU‐CCS‐1 cells at a much higher dose, with an IC50 of 2μM (Figure 4B). When we treated both cell lines with 1μM of AZD6738 for 3 days, significantly reduced viability was seen in SU‐CCS‐1 cells (14%) compared to U2OS cells (56%, Figure 4C, *p* < .0001). When we combined drug treatment with radiation, all three treatments significantly inhibit tumour cell growth, however, only the combination of radiation with AZD6738 significantly reduced tumour cell numbers (Figure 4D, *p* < .0001). Overall, these results suggested that ATR inhibition can induce a strong synthetic lethal effect in CCS cells. The limitation of this study is that we have not been able to obtain more CCS cell lines or use animal models for the experiment.

**FIGURE 4:**
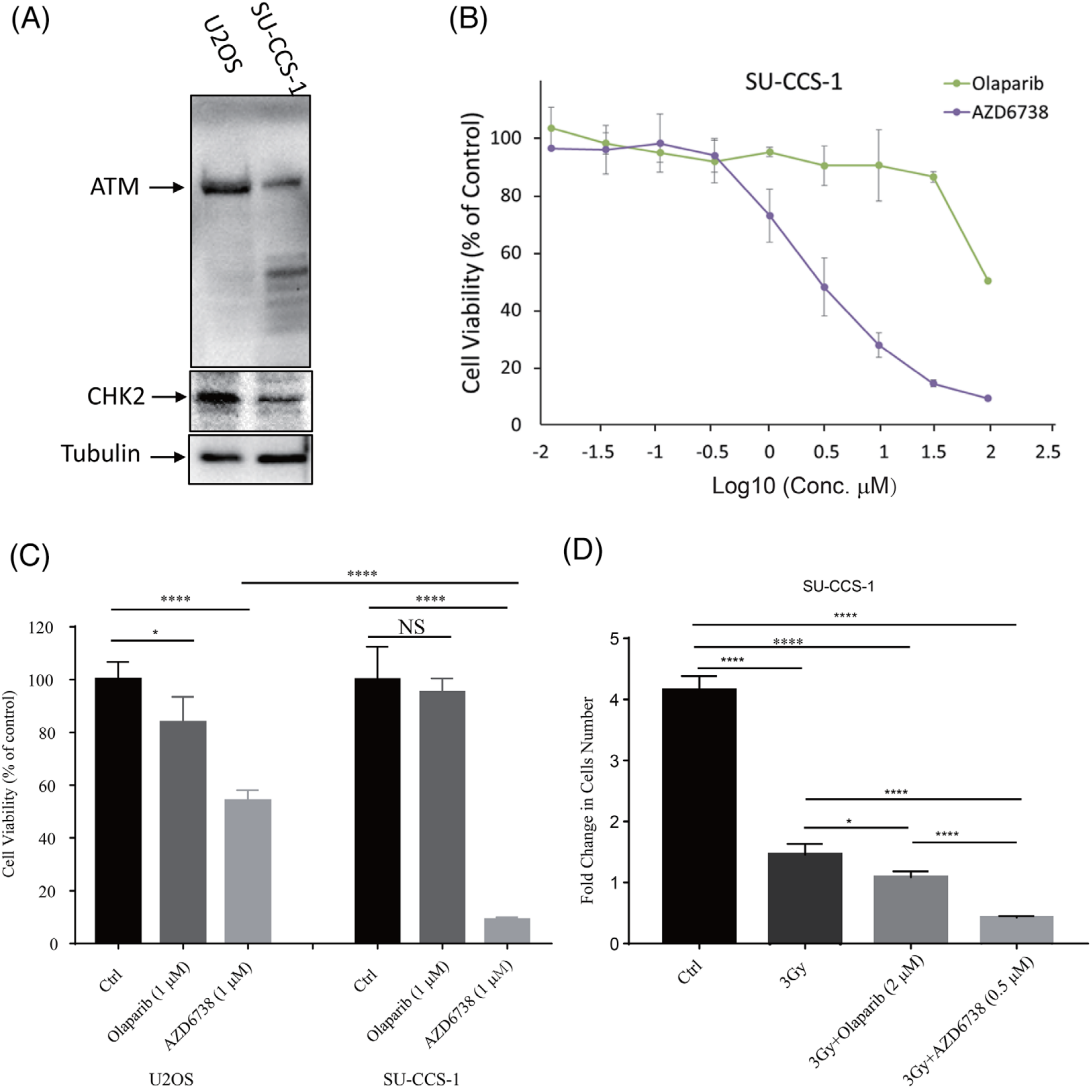
Clear cell carcoma (CCS) cell line‐SU‐CCS‐1 is sensitive to ATR inhibition. (A) ATM alteration is associated with metastatic disease in CCS (Fisher's exact test, p = 0.0147); (B) western blot (WB) of ATM level in CCS cell line (SU‐CCS‐1) vs. osteosarcoma cell line (U2OS); (C) SU‐CCS‐1 cells were treated with two selective inhibitors targeting ATR (AZD6738) and PARP (Olaparib), and cell viability assay showed that SU‐CCS‐1 is more sensitive to ATR inhibition than PARP inhibition; (D) Five days after treatment, cell viability assay showed that there are much less viable SU‐CCS‐1 cells when ATR was inhibited. (Student *t*‐test, * *p* < .05, **** *p* < .0001)

In summary, our study provides the first comprehensive view of the genomic alterations of CCS. Our study also suggests that CCS patients with *ATM* alterations may be likely to benefit from treatment with an ATR inhibitor.

## CONFLICT OF INTEREST

The authors declare that they have no conflict of interest.

## FUNDING INFORMATION

This work is supported by the National Natural Science Foundation of China (Nos. 81702826, 81772910 and 81802725), the Natural Science Foundation of Guangdong Province, China (No. 2019A1515011263), and the Shenzhen Municipal Government of China (No. 20170731162715261).

## Supporting information



Supporting Information

Supporting Information
