# Supplementary material for: Whole‐exome sequencing in clear cell sarcoma of soft tissue uncovers novel prognostic categorization and drug targets
Source: Clin Transl Med. 2021 Dec 19;11(12):e640. doi: 10.1002/ctm2.640 (PMC8684775; doi:10.1002/ctm2.640)
Supplement: Supplementary file 1 — Supporting Information [file CTM2-11-e640-s001.docx]

**Whole-exome Sequencing in Clear Cell Sarcoma of Soft Tissue Uncovers Novel Prognostic Categorization and Drug Targets**

Jingjing Li,^1,3,4, #^ Chao Chen,^2,5, #^ Wei Liu,^1,3, 4, #^ Songming Liu,^2,5^ Wanming Hu,^3,4,6^ Xiaoyan Gao, ^2^ Geng Liu,^2^ Dandan Li,^1, 3,4^ Ya Ding,^1, 3,4^ Xizhi Wen, ^1, 3,4^ Xiuqing Zhang,^2,5^ Yong Hou, ^2,5, *^ Xing Zhang, ^1,3,4, *^ Bo Li, ^2, *^ Xiaoshi Zhang,^1,3,4, *^ Xi Zhang^2,5, *^

^1^ Biotherapy Center, Sun Yat-sen University Cancer Center, 510060 Guangzhou, China.

^2^ BGI-Shenzhen, Shenzhen, 518083, China.

^3^ State Key Laboratory of Oncology in South China,510060 Guangzhou, China.

^4^ Collaborative Innovation Center for Cancer Medicine, 510060 Guangzhou, China.

^5^ China National GeneBank, BGI-Shenzhen, Shenzhen 518120, China.

^6^ Department of pathology, Sun Yat-sen University Cancer Center, 510060 Guangzhou, China.

# These authors contributed equally.

* **Correspondence:** zhangxi1@genomics.cn; zhangxsh@sysucc.org.cn; libo@genomics.cn; zhangxing@sysucc.org.cn; houyong@genomics.cn.

**Supplementary data**


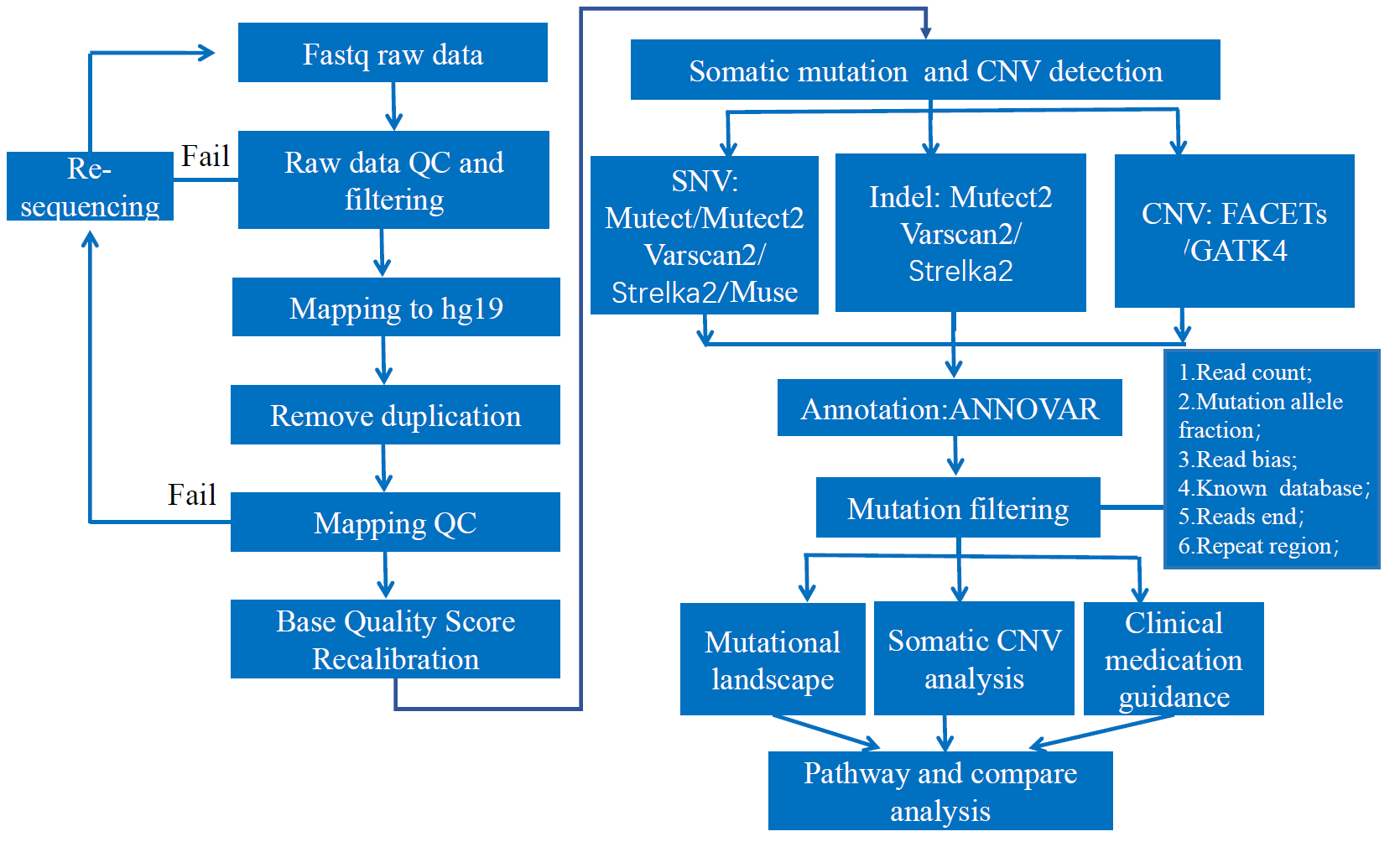


**Figure S1. The Bioinformatics analysis pipeline of CCS (N=21).** Data filtering and mapping are shown on the left, and mutation detection and statistical analysis are shown on the right.


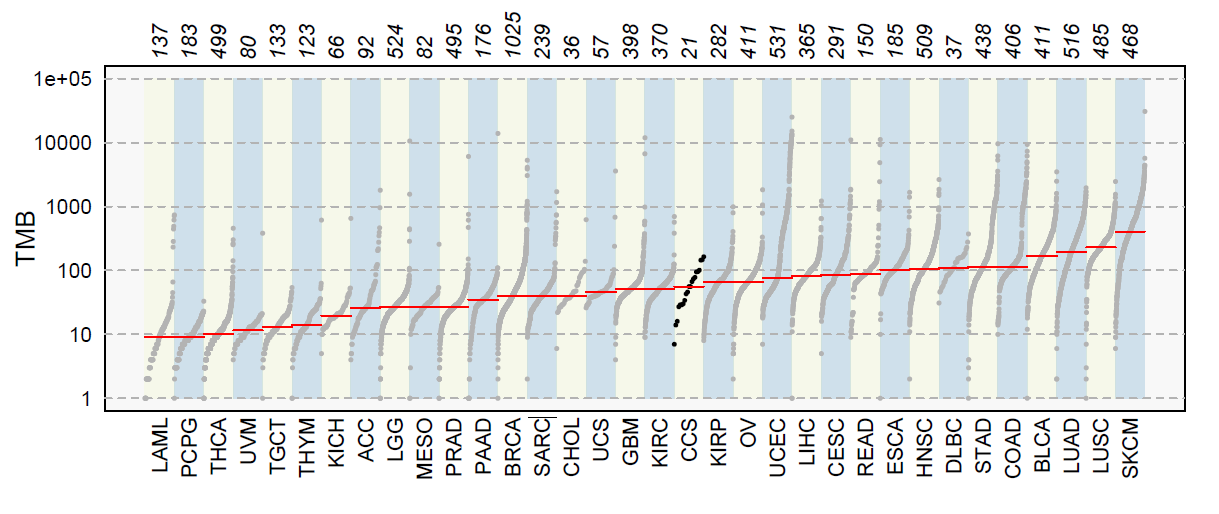


**Figure S2. Comparison of CCS Mutation Load with Other TCGA Tumor Types.** The TMB of CCS is low and comparable with TCGA SARC cohort.


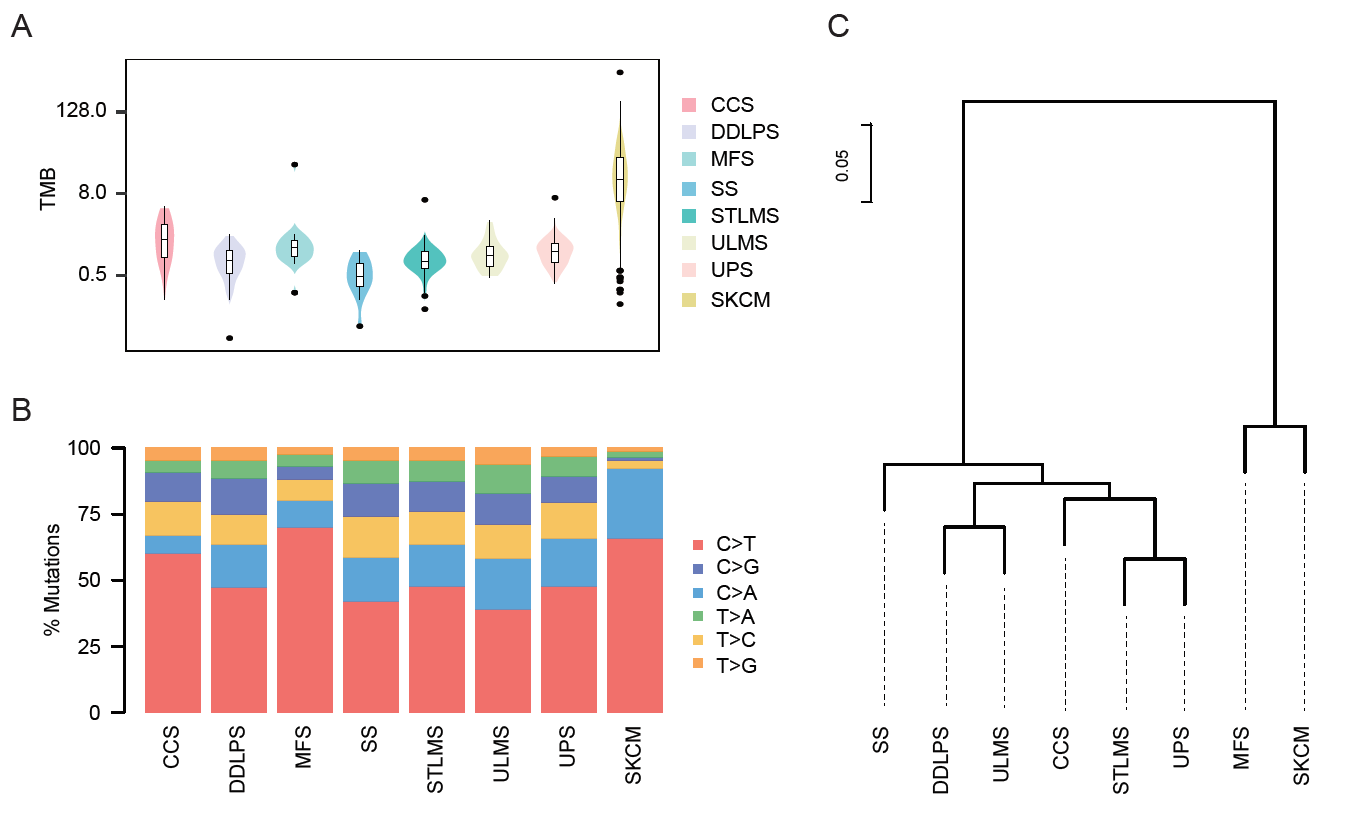

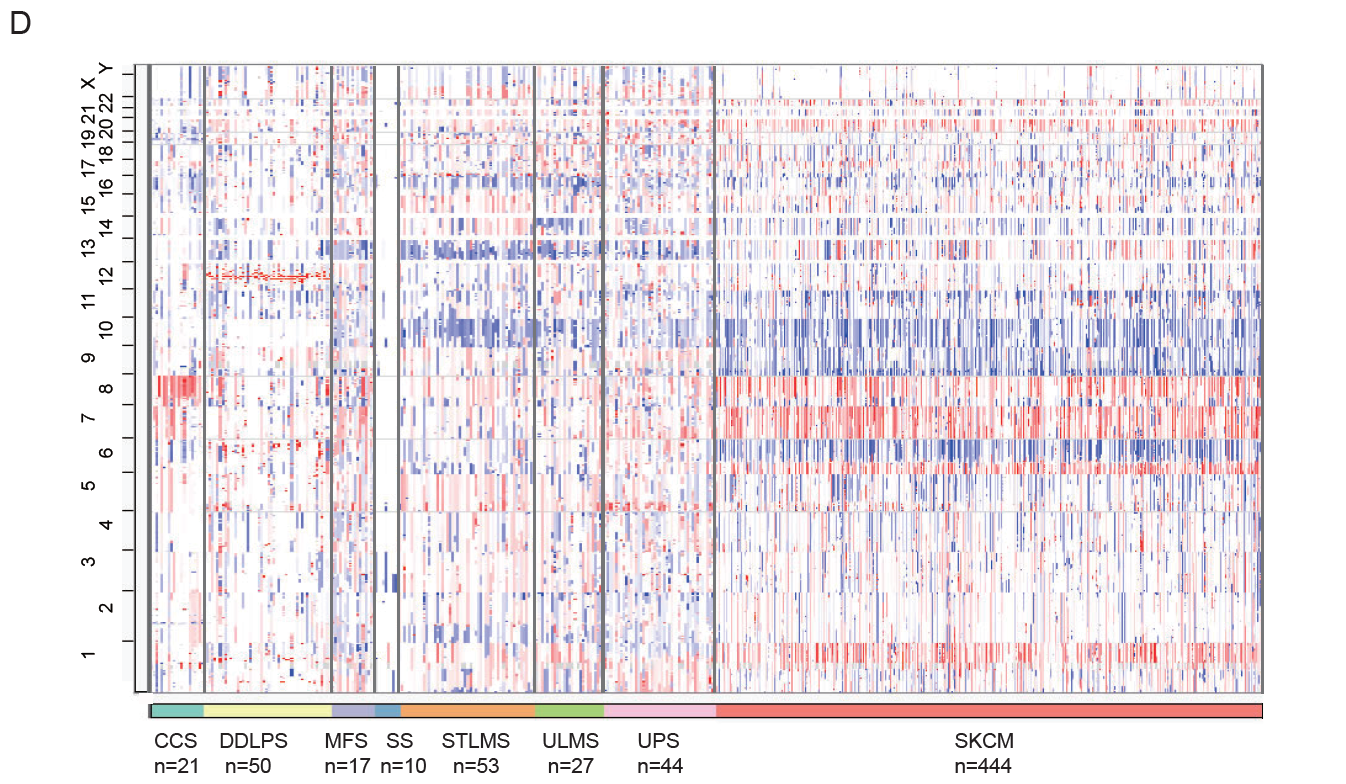


**Figure S3.** **Comparison between CCS (N=21), other subtypes of sarcomas, and melanoma.** **A** Distribution of TMB of CCS and other cancer types. **B** Comparison of six mutation types between CCS and other associated cancers. **C** Clustering of mutational signatures based on six mutation types and 16 types of nucleotides flanking the mutated base for each cancer type. **D** CCS is compared at CNV level with other sarcomas and skin cutaneous melanoma (SKCM).


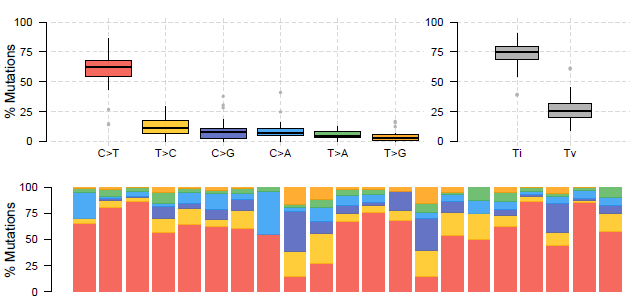

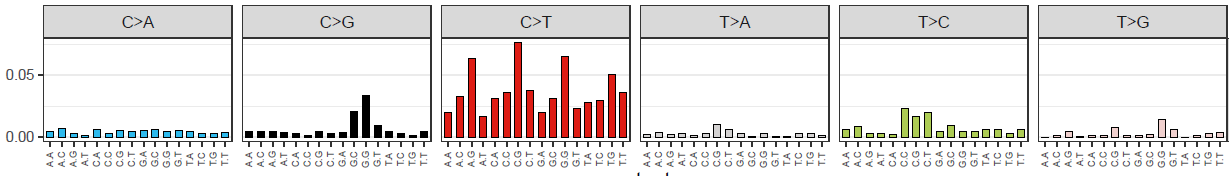


**Figure S4. Mutation spectrum analysis of CCS.** Mutation spectrum is calculated from 21 CCS samples sequenced by WES.


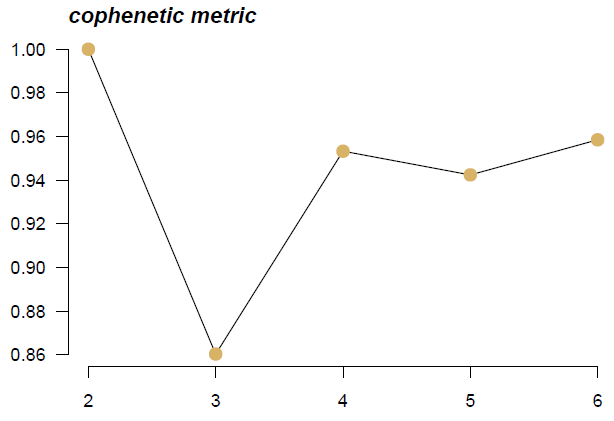


**Figure S5. The optimal number of signatures were estimated.** The optimal number of signatures was estimated with function ‘plotCophenetic’ in maftools using Bayes NMF algorithms.


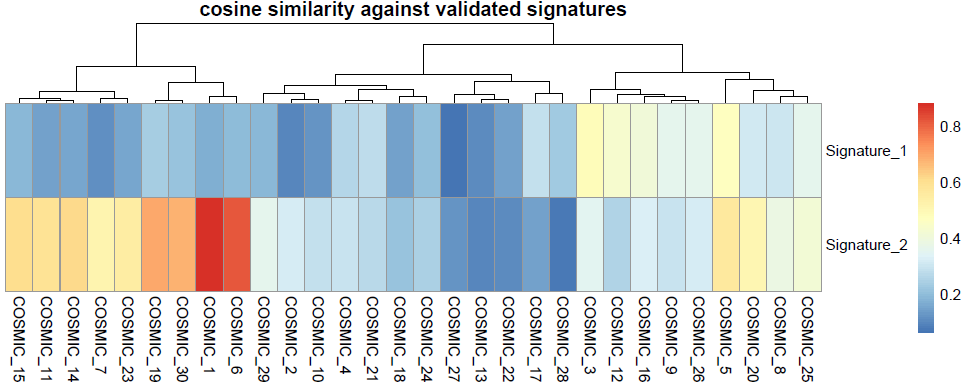


**Figure S6. Two stable mutation signatures found in CCS.** Signature1, best match: COSMIC_1 (cosine-similarity: 0.884); Signature2, best match: COSMIC_3 (cosine-similarity: 0.489).


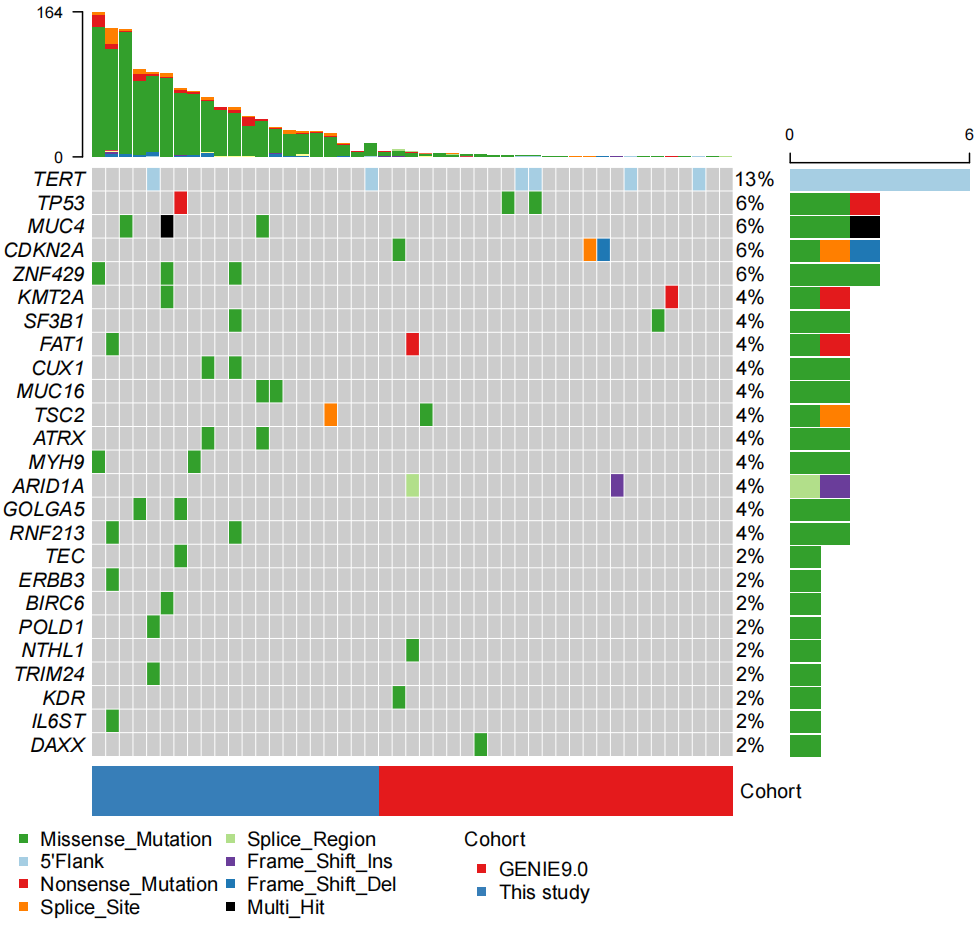


**Figure S7. TOP25 mutated cancer-related genes in CCS from this study and GENIE9.0.** The left panel shows 21 CCS samples in this study (WES sequencing), the right panel shows 26 CCS samples in GENIE9.0 (gene panel).


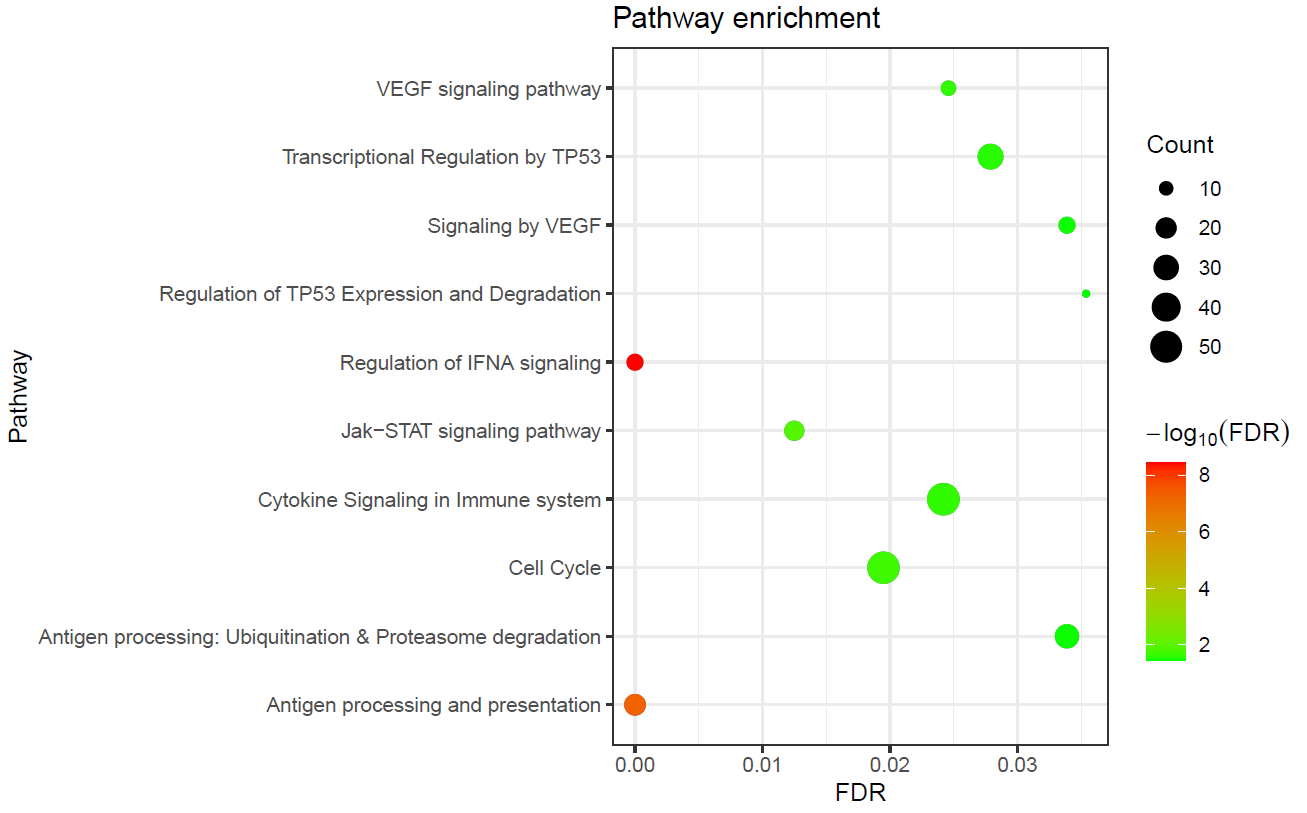


**Figure S8. Enriched pathways of genes in somatic CNV regions.** The genes in deletion or amplification regions were used as input of GSEA, the representative pathway terms were selected and visualized.


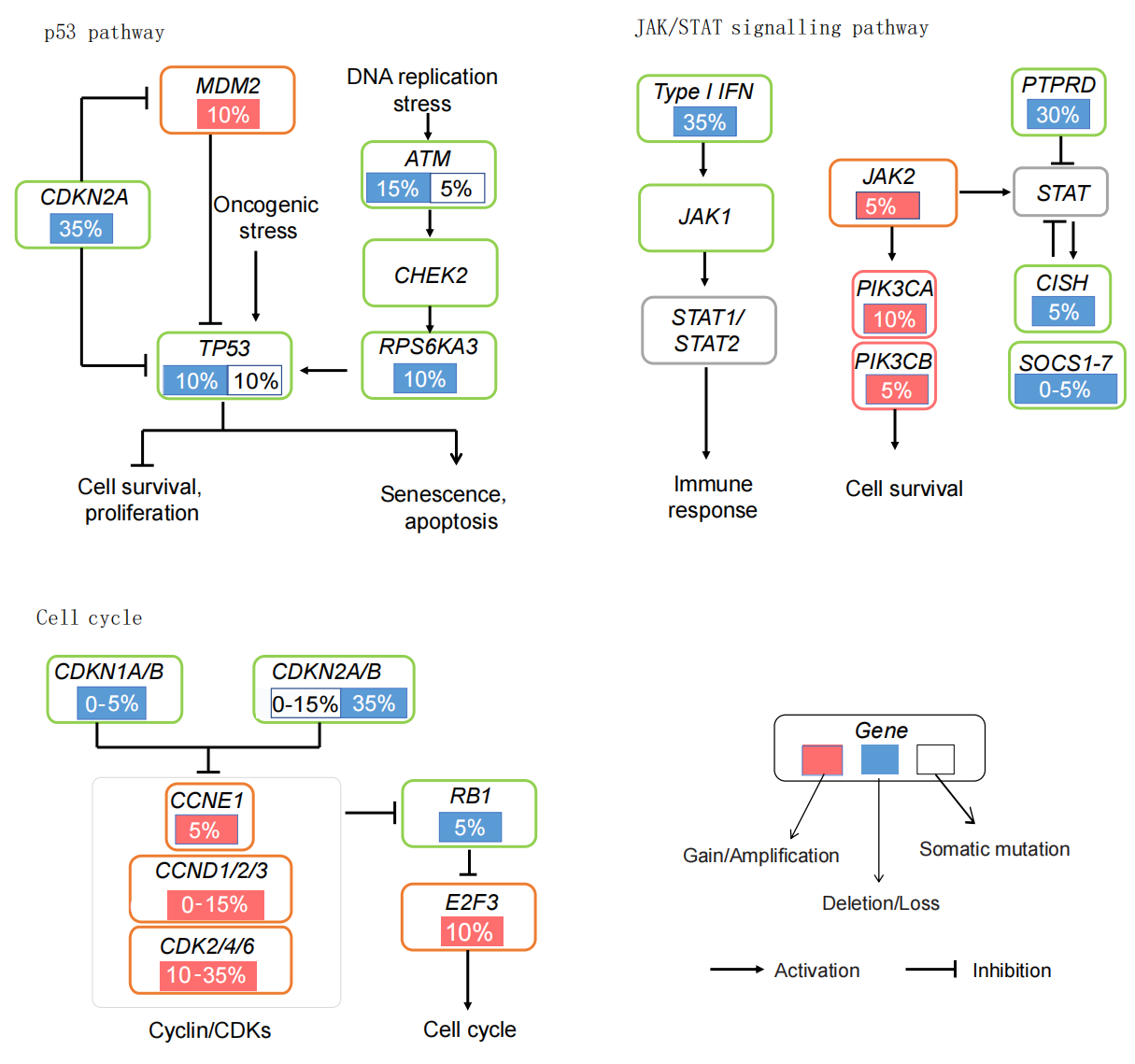


**Figure S9. Somatic CNV of three pathways in the GENIE CCS cohort (N=20). A** p53 pathway. **B** JAK/STAT signalling pathway. **C** cell cycle pathway.


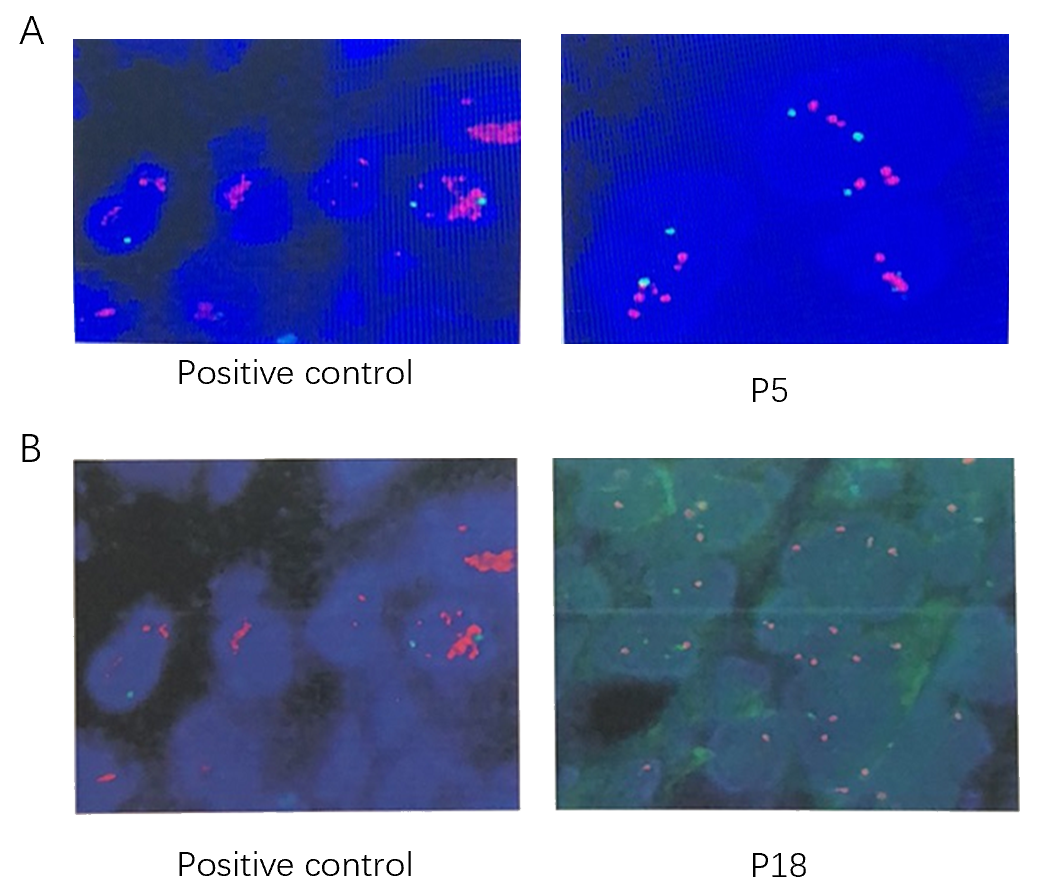


**Figure S10**. **Amplification status of MDM2 was confirmed by FISH in two tumor samples.** P5 is positive (**A**) and P18 is negative (**B**).


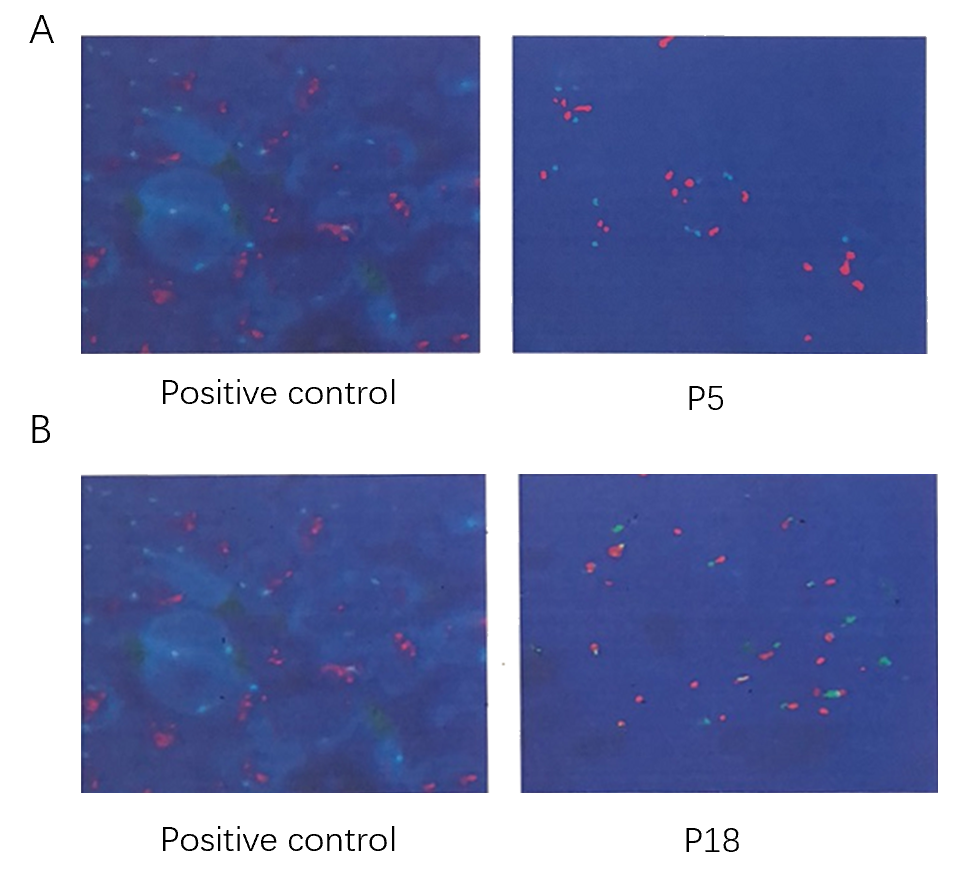


**Figure S11**. **Amplification status of CDK4 was confirmed by FISH in two tumor samples.** P5 is positive (**A**) and P18 is negative (**B**).


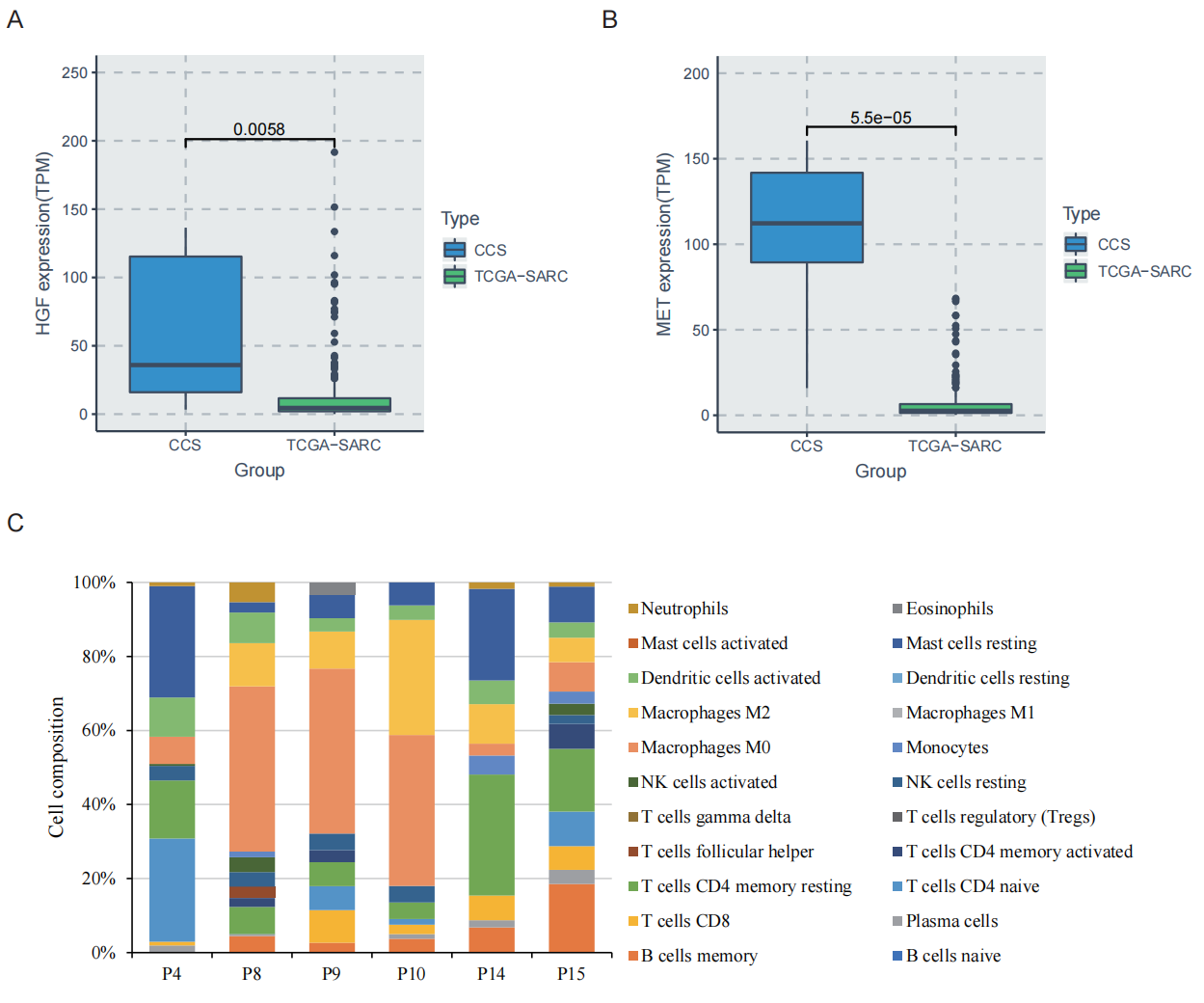


**Figure S12. RNA-seq analysis of 6 CCS samples.** The gene expression of HGF(A), MET(B), and Proportion of immune cells (C) predicted by gene expressions.


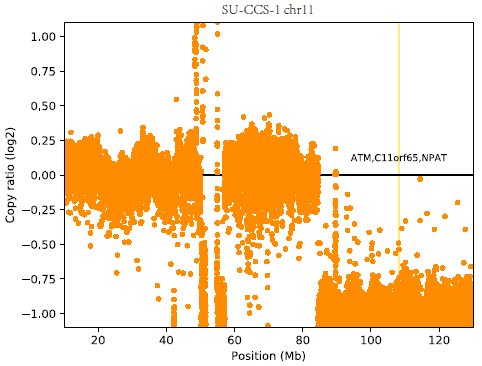


**Figure S13. CNV pattern of SU-CCS-1 cell line.** It shows copy number deletion at ATM and adjacent genes.

**Table S1. Clinical information of the CCS patients.**

| Case ID | Age | Stage | Sex | Primary Site | EWSR1 Fusion | PD-L1 IHC | RFI.time | RFI.status | OS.time | OS.status | Tumor Purity | Tumor ploidy | 9p21.3 Deletion |
| --- | --- | --- | --- | --- | --- | --- | --- | --- | --- | --- | --- | --- | --- |
| P1 | 44 | I | F | Foot | EWSR1-ATF1 | neg | 20 | 1 | 44 | 1 | 0.316 | 0.82 | N |
| P2 | 32 | I | M | Popliteal fossa | EWSR1-ATF1 | neg | 3.5 | 1 | 12 | 0 | 0.445 | 1.607 | Y |
| P3 | 49 | I | M | Leg | EWSR1-ATF1 | ni | 1.5 | 1 | 14 | 1 | 0.719 | 3.482 | Y |
| P4 | 41 | I | F | Infrapatellar | EWSR1-ATF1 | neg | 48.5 | 0 | 67.5 | 0 | 0.3 | 2.148 | N |
| P5 | 58 | IV | F | Peritoneal | EWSR1-ATF1 | ni | 18 | 1 | 18 | 1 | 0.602 | 2.507 | N |
| P6 | 40 | II | F | Foot | EWSR1 translocation | neg | 21 | 0 | 29 | 0 | 0.811 | 1.792 | N |
| P7 | 21 | I | M | Knee | EWSR1-ATF1 | ni | 63 | 1 | 63 | 1 | 0.744 | 1.812 | N |
| P8 | 25 | I | F | Hand | EWSR1-ATF1 | neg | 27 | 1 | 33 | 0 | 0.315 | 1.45 | N |
| P9 | 22 | I | F | Hip | EWSR1 translocation | ni | 1 | 1 | 8 | 1 | 0.654 | 3.818 | N |
| P10 | 27 | IV | M | Elbow joint | EWSR1 translocation | ni | 2.5 | 1 | 2.5 | 1 | 0.812 | 1.866 | Y |
| P11 | 26 | I | M | Leg | EWSR1-ATF1 | pos | 6 | 1 | 31 | 1 | 0.539 | 3.294 | N |
| P12 | 20 | IV | M | Elbow joint | EWSR1 translocation | ni | 7 | 1 | 33 | 1 | 0.818 | 2.249 | N |
| P13 | 24 | I | F | Elbow joint | EWSR1-ATF1 | neg | 11 | 1 | 72 | 0 | 0.657 | 1.788 | N |
| P14 | 52 | I | M | Foot | EWSR1-ATF1 | ni | 117 | 1 | 134 | 0 | 0.338 | 1.739 | Y |
| P15 | 27 | I | F | Foot | EWSR1 translocation | ni | 16 | 1 | 45 | 0 | 0.61 | 1.901 | N |
| P16 | 20 | I | F | Hand | EWSR1-ATF1 | ni | 20 | 1 | 138 | 0 | 0.331 | 1.56 | N |
| P17 | 40 | I | F | Ankle joint | EWSR1-ATF1 | neg | 32 | 0 | 32 | 0 | 0.26 | 2.393 | Y |
| P18 | 47 | I | F | Foot | EWSR1 translocation | neg | 120 | 1 | 192 | 1 | 0.344 | 2.742 | N |
| P19 | 34 | I | M | Foot | EWSR1 translocation | ni | 9 | 0 | 9 | 0 | 0.3 | 1.339 | Y |
| P20 | 37 | I | M | Foot | EWSR1-ATF1 | ni | 5 | 1 | 26 | 1 | 0.799 | 2.07 | Y |
| P21 | 21 | II | F | Foot | EWSR1-ATF1 | neg | 1 | 1 | 36 | 0 | 0.351 | 1.612 | N |

Note: F, female; M, male; Neg, negative; ni, not identified; pos, positive; EWSR1-ATF1, EWS RNA binding protein 1-Activating Transcription Factor 1; PD-L1, programmed death-ligand 1; RFI, recurrence free interval; OS, overall survival.

**Table S2. WES sequencing coverage and depth of each sample.**

| Sample | Raw data (Gb) | Mapping Rate | Target Rate | Coverage (rmdup) | Coverage-0X | Coverage-4X | Coverage-10X | Coverage-30X | Coverage-100X |
| --- | --- | --- | --- | --- | --- | --- | --- | --- | --- |
| P1-Tumor | 87.27 | 97.99% | 42.70% | 157.87 | 99.75% | 99.29% | 98.22% | 94.65% | 83.85% |
| P2-Tumor | 68.24 | 98.38% | 63.03% | 164.76 | 99.90% | 99.75% | 99.33% | 97.87% | 91.67% |
| P3-Tumor | 54.79 | 98.38% | 58.53% | 144.65 | 99.90% | 99.73% | 99.26% | 97.34% | 87.44% |
| P4-Tumor | 113.05 | 98.91% | 58.80% | 261.17 | 99.89% | 99.78% | 99.56% | 98.74% | 95.59% |
| P5-Tumor | 67.48 | 99.36% | 73.52% | 218.46 | 99.76% | 99.43% | 98.85% | 97.17% | 90.86% |
| P6-Tumor | 23.1 | 99.01% | 78.85% | 81.9 | 99.56% | 99.22% | 98.38% | 94.66% | 76.22% |
| P7-Tumor | 7.37 | 99.74% | 76.90% | 58.1 | 97.98% | 96.40% | 93.65% | 79.93% | 28.41% |
| P8-Tumor | 70.64 | 98.73% | 48.81% | 293.09 | 99.86% | 99.51% | 98.64% | 95.78% | 85.33% |
| P9-Tumor | 166.79 | 99.80% | 73.79% | 593.65 | 99.83% | 99.72% | 99.52% | 99.06% | 97.32% |
| P10-Tumor | 23.01 | 99.84% | 72.96% | 80.25 | 99.41% | 98.50% | 96.78% | 90.83% | 69.99% |
| P11-Tumor | 102.1 | 98.61% | 50.89% | 294.98 | 99.95% | 99.82% | 99.42% | 97.94% | 92.56% |
| P12-Tumor | 76.91 | 99.44% | 60.75% | 107.62 | 99.71% | 99.44% | 98.82% | 96.67% | 87.67% |
| P13-Tumor | 53.52 | 99.41% | 73.54% | 318.06 | 99.76% | 99.47% | 98.95% | 97.17% | 90.11% |
| P14-Tumor | 52.07 | 99.80% | 77.71% | 284.29 | 99.87% | 99.62% | 99.26% | 98.04% | 92.61% |
| P15-Tumor | 90.34 | 99.85% | 80.22% | 144.85 | 99.50% | 99.26% | 98.90% | 98.00% | 94.74% |
| P16-Tumor | 86.44 | 98.76% | 47.43% | 351 | 99.86% | 99.72% | 99.34% | 97.89% | 92.35% |
| P17-Tumor | 94.49 | 98.99% | 74.30% | 268.87 | 99.78% | 99.58% | 99.23% | 98.19% | 94.23% |
| P18-Tumor | 121.82 | 98.15% | 63.20% | 183.73 | 99.73% | 99.40% | 98.80% | 97.02% | 90.91% |
| P19-Tumor | 139.75 | 99.84% | 76.65% | 231.31 | 99.95% | 99.87% | 99.67% | 99.14% | 96.98% |
| P20-Tumor | 152.02 | 98.36% | 44.81% | 390.94 | 99.58% | 99.16% | 98.54% | 96.85% | 90.90% |
| P21-Tumor | 214.57 | 99.03% | 72.62% | 311.76 | 99.89% | 99.81% | 99.69% | 99.35% | 98.32% |
| P1-Normal | 34.8 | 99.28% | 50.39% | 226.43 | 99.81% | 99.53% | 98.82% | 95.62% | 77.65% |
| P2-Normal | 33.23 | 99.30% | 49.37% | 209.51 | 99.93% | 99.65% | 98.81% | 95.32% | 76.26% |
| P3-Normal | 32.41 | 99.29% | 49.68% | 206.95 | 99.92% | 99.68% | 98.94% | 95.51% | 75.84% |
| P4-Normal | 14.74 | 99.13% | 38.37% | 72.87 | 99.69% | 98.89% | 96.69% | 83.54% | 26.33% |
| P5-Normal | 35.05 | 99.39% | 49.53% | 220.04 | 99.76% | 99.44% | 98.55% | 95.07% | 77.09% |
| P6-Normal | 20.59 | 98.24% | 54.85% | 149.74 | 99.73% | 99.05% | 97.27% | 89.53% | 58.82% |
| P7-Normal | 41.28 | 99.29% | 48.69% | 93.79 | 99.80% | 99.52% | 98.84% | 96.22% | 82.70% |
| P8-Normal | 41.08 | 99.35% | 47.75% | 247.44 | 99.78% | 99.58% | 98.96% | 96.15% | 80.45% |
| P11-Normal | 20.34 | 99.39% | 74.40% | 188.47 | 99.78% | 99.26% | 98.17% | 94.26% | 73.99% |
| P12-Normal | 30.68 | 99.51% | 53.02% | 171.97 | 99.93% | 99.69% | 99.02% | 96.10% | 78.00% |
| P13-Normal | 24.58 | 99.36% | 51.63% | 164.63 | 99.71% | 99.27% | 98.14% | 92.85% | 66.52% |
| P14-Normal | 78.46 | 99.53% | 57.20% | 313.37 | 99.94% | 99.84% | 99.61% | 98.91% | 95.09% |
| P15-Normal | 45.81 | 99.38% | 50.52% | 105.35 | 99.66% | 99.40% | 98.80% | 96.55% | 85.51% |
| P16-Normal | 36.24 | 99.37% | 53.14% | 243.05 | 99.80% | 99.59% | 98.88% | 95.60% | 78.35% |
| P17-Normal | 24.83 | 99.14% | 41.70% | 126.19 | 99.62% | 98.92% | 97.53% | 91.95% | 59.15% |
| P18-Normal | 21.82 | 99.43% | 45.17% | 105.81 | 99.72% | 99.14% | 97.67% | 91.48% | 55.92% |
| P19-Normal | 40.2 | 99.50% | 49.87% | 106.68 | 99.86% | 99.64% | 99.13% | 96.93% | 83.89% |
| P20-Normal | 101.95 | 98.77% | 50.55% | 464.3 | 99.85% | 99.61% | 99.18% | 98.20% | 95.28% |
| P21-Normal | 32.76 | 99.33% | 51.96% | 212.56 | 99.78% | 99.54% | 98.77% | 95.27% | 75.02% |

**Table S3. MAF file of the CCS samples.** This file is provided separately in Excel format.

**Table S4. The mutation signatures of CCS.**

| Signature | Proposed Etiology | All | P1 | P2 | P3 | P4 | P5 | P6 | P7 | P8 | P9 | P10 | P11 | P12 | P13 | P14 | P15 | P16 | P17 | P18 | P19 | P20 | P21 |
| --- | --- | --- | --- | --- | --- | --- | --- | --- | --- | --- | --- | --- | --- | --- | --- | --- | --- | --- | --- | --- | --- | --- | --- |
| 1 | Age | 0.419 | 0.138 | 0.337 | 0.78 | 0.832 | 0.783 | 0.428 | 0.131 | 0.258 | 0.002 | 0.057 | 0.165 | 0.208 | 0.628 | 0.041 | 0.215 | 0.72 | 0.188 | 0.434 | 0 | 0.671 | 0.377 |
| 2 | APOBEC | 0.036 | 0.023 | 0.081 | 0.004 | 0 | 0.036 | 0.047 | 0 | 0 | 0 | 0 | 0.085 | 0.023 | 0 | 0.004 | 0.082 | 0 | 0 | 0.07 | 0.034 | 0 | 0.04 |
| 3 | BRCA1 / BRCA2 (failure of DNA DSBR / large INDELs) | 0.135 | 0 | 0 | 0 | 0 | 0.013 | 0 | 0 | 0 | 0.602 | 0.248 | 0.051 | 0 | 0 | 0.535 | 0.176 | 0 | 0 | 0 | 0.12 | 0 | 0 |
| 4 | Smoking | 0 | 0.073 | 0 | 0 | 0 | 0 | 0 | 0 | 0 | 0 | 0 | 0 | 0.029 | 0 | 0 | 0 | 0 | 0 | 0 | 0 | 0 | 0 |
| 5 | Unknown (all cancer types) | 0 | 0 | 0 | 0 | 0 | 0 | 0 | 0 | 0 | 0 | 0 | 0 | 0 | 0 | 0 | 0 | 0 | 0 | 0 | 0 | 0 | 0 |
| 6 | Defective DNA MMR / MSI (small INDELs) | 0.132 | 0.462 | 0.295 | 0 | 0 | 0 | 0.115 | 0.149 | 0.322 | 0 | 0 | 0.414 | 0.034 | 0 | 0 | 0.026 | 0 | 0.477 | 0.003 | 0.165 | 0.16 | 0 |
| 7 | UV light | 0.043 | 0 | 0 | 0.092 | 0 | 0 | 0 | 0.175 | 0.02 | 0 | 0.07 | 0 | 0.016 | 0 | 0 | 0 | 0 | 0 | 0.047 | 0.137 | 0.068 | 0 |
| 8 | Unknown (breast cancer and medulloblastoma) | 0 | 0 | 0 | 0 | 0 | 0.028 | 0 | 0 | 0 | 0 | 0 | 0 | 0 | 0 | 0 | 0 | 0 | 0 | 0 | 0 | 0 | 0 |
| 9 | POLH (CLL, BCL) | 0 | 0 | 0 | 0 | 0 | 0 | 0 | 0 | 0 | 0 | 0 | 0 | 0 | 0 | 0 | 0 | 0 | 0 | 0 | 0 | 0 | 0 |
| 10 | POLE (ultra-hypermutation) | 0 | 0 | 0.012 | 0 | 0 | 0 | 0 | 0.054 | 0 | 0 | 0.033 | 0.037 | 0 | 0 | 0 | 0 | 0 | 0 | 0 | 0.088 | 0.012 | 0 |
| 11 | Alkylating agents | 0.073 | 0.211 | 0 | 0.068 | 0 | 0.052 | 0.066 | 0.079 | 0 | 0 | 0 | 0.103 | 0.112 | 0.261 | 0.024 | 0.167 | 0 | 0 | 0 | 0 | 0 | 0.086 |
| 12 | Unknown (liver cancer) | 0.037 | 0 | 0 | 0 | 0 | 0.06 | 0 | 0.043 | 0 | 0.186 | 0.323 | 0 | 0 | 0 | 0.177 | 0.219 | 0 | 0 | 0 | 0 | 0 | 0.004 |
| 13 | APOBEC | 0 | 0 | 0 | 0 | 0.025 | 0 | 0.038 | 0.019 | 0 | 0 | 0 | 0.01 | 0 | 0 | 0 | 0 | 0 | 0 | 0 | 0.056 | 0 | 0 |
| 14 | Unknown (uterine cancer and glioma / hypermutation) | 0 | 0 | 0 | 0 | 0 | 0 | 0 | 0 | 0 | 0 | 0 | 0 | 0 | 0 | 0 | 0 | 0 | 0 | 0 | 0 | 0 | 0 |
| 15 | Defective DNA MMR (small INDELs) | 0 | 0 | 0 | 0 | 0.022 | 0 | 0 | 0.215 | 0 | 0.014 | 0 | 0.109 | 0 | 0 | 0.018 | 0 | 0 | 0 | 0 | 0 | 0 | 0.332 |
| 16 | Unknown (liver cancer) | 0 | 0 | 0 | 0 | 0 | 0 | 0 | 0.066 | 0 | 0 | 0 | 0 | 0 | 0 | 0 | 0 | 0 | 0 | 0 | 0 | 0 | 0 |
| 17 | Unknown (different cancers) | 0.029 | 0 | 0 | 0 | 0.028 | 0.014 | 0.018 | 0.018 | 0 | 0.083 | 0.085 | 0 | 0 | 0 | 0.139 | 0.036 | 0.009 | 0 | 0 | 0.021 | 0 | 0 |
| 18 | Unknown (different cancers) | 0 | 0.046 | 0 | 0 | 0 | 0 | 0 | 0 | 0.243 | 0 | 0 | 0 | 0 | 0 | 0 | 0 | 0 | 0 | 0 | 0 | 0 | 0 |
| 19 | Unknown (pilocytic astrocytoma) | 0 | 0 | 0 | 0.037 | 0 | 0 | 0.105 | 0 | 0 | 0 | 0 | 0 | 0.117 | 0 | 0 | 0 | 0 | 0 | 0.022 | 0 | 0 | 0 |
| 20 | Defective DNA MMR (small INDELs) | 0 | 0 | 0 | 0 | 0 | 0 | 0 | 0 | 0 | 0.104 | 0.047 | 0 | 0 | 0 | 0 | 0 | 0 | 0.045 | 0 | 0.119 | 0 | 0 |
| 21 | Unknown (stomach cancer / MSI) | 0 | 0 | 0 | 0 | 0 | 0 | 0 | 0 | 0 | 0 | 0 | 0 | 0 | 0 | 0.005 | 0.004 | 0.094 | 0.068 | 0 | 0.069 | 0 | 0 |
| 22 | Aristolochic acid | 0.022 | 0 | 0.037 | 0 | 0.093 | 0 | 0.006 | 0.051 | 0 | 0 | 0.025 | 0.026 | 0 | 0 | 0.056 | 0 | 0.028 | 0 | 0 | 0.039 | 0.006 | 0.128 |
| 23 | Unknown (liver cancer) | 0.071 | 0 | 0.195 | 0.019 | 0 | 0.014 | 0.008 | 0 | 0 | 0 | 0.046 | 0 | 0.017 | 0.052 | 0 | 0.075 | 0 | 0.077 | 0.095 | 0.124 | 0.083 | 0 |
| 24 | Aflatoxin | 0 | 0 | 0 | 0 | 0 | 0 | 0 | 0 | 0 | 0 | 0.039 | 0 | 0 | 0 | 0 | 0 | 0.118 | 0.024 | 0 | 0.028 | 0 | 0.02 |
| 25 | Unknown (Hodgkin lymphoma) | 0 | 0 | 0 | 0 | 0 | 0 | 0 | 0 | 0 | 0 | 0 | 0 | 0.087 | 0 | 0 | 0 | 0 | 0 | 0 | 0 | 0 | 0 |
| 26 | Defective DNA MMR (small INDELs) | 0 | 0 | 0 | 0 | 0 | 0 | 0 | 0 | 0 | 0 | 0 | 0 | 0 | 0.058 | 0 | 0 | 0.029 | 0 | 0 | 0 | 0 | 0 |
| 27 | Unknown (kidney clear cell carcinomas / small INDELs) | 0 | 0.006 | 0 | 0 | 0 | 0 | 0 | 0 | 0 | 0 | 0 | 0 | 0 | 0 | 0 | 0 | 0 | 0 | 0.003 | 0 | 0 | 0.008 |
| 28 | Unknown (stomach cancer) | 0 | 0 | 0 | 0 | 0 | 0 | 0 | 0 | 0 | 0.009 | 0 | 0 | 0 | 0 | 0 | 0 | 0.002 | 0 | 0 | 0 | 0 | 0.005 |
| 29 | Tobacco chewing | 0 | 0.04 | 0 | 0 | 0 | 0 | 0.131 | 0 | 0.157 | 0 | 0.028 | 0 | 0 | 0 | 0 | 0 | 0 | 0 | 0 | 0 | 0 | 0 |
| 30 | Unknown (breast cancer) | 0.003 | 0 | 0.044 | 0 | 0 | 0 | 0.038 | 0 | 0 | 0 | 0 | 0 | 0.358 | 0 | 0 | 0 | 0 | 0.122 | 0.324 | 0 | 0 | 0 |

**Table S5. Recurrent mutated genes of CCS and their mutation frequency.**

| Gene Symbol | CGC | Frequency |
| --- | --- | --- |
| AGRN | . | 2 |
| AHNAK2 | . | 2 |
| ATRX | pancreatic neuroendocrine tumors, pediatric GBM, TSG | 2 |
| B4GALNT4 | . | 2 |
| BRS3 | . | 2 |
| BTBD2 | . | 2 |
| CADPS | . | 2 |
| CCDC157 | . | 2 |
| CCDC40 | . | 2 |
| CDH4 | . | 2 |
| CENPE | . | 2 |
| CLIC6 | . | 2 |
| CNPY4 | . | 2 |
| CUX1 | endometrial, melanoma, colorectal, AML, MDS, other tumor types, oncogene, TSG | 2 |
| DBN1 | . | 2 |
| DCHS1 | . | 2 |
| DGKD | . | 2 |
| DMD | . | 2 |
| DPPA3 | . | 2 |
| DUSP26 | . | 2 |
| DUSP27 | . | 2 |
| DYSF | . | 2 |
| FBN3 | . | 2 |
| FLG2 | . | 2 |
| FLT1 | . | 2 |
| GABRA5 | . | 2 |
| GAK | . | 2 |
| GDF10 |  | 2 |
| GHDC | . | 2 |
| GOLGA5 | papillary thyroid, Spitzoid tumor, fusion | 2 |
| HDAC6 | . | 2 |
| HDAC7 | . | 2 |
| HECW2 | . | 2 |
| HOXD8 | . | 2 |
| HVCN1 | . | 2 |
| IL2RB | . | 2 |
| KCNG1 | . | 2 |
| KCNH2 | . | 2 |
| KCNMA1 | . | 2 |
| KCNS1 | . | 2 |
| KIAA0100 | . | 2 |
| KNDC1 | . | 2 |
| KRT76 | . | 2 |
| LTBP3 | . | 2 |
| MAGEC1 | . | 2 |
| MAGEL2 | . | 2 |
| MAP4 | . | 2 |
| MAPK8IP3 | . | 2 |
| MAST1 | . | 2 |
| MRPL1 | . | 2 |
| MUC16 | HNSCC, melanoma, oncogene | 2 |
| MUC4 | HNSCC, oncogene | 3 |
| MYH14 | . | 2 |
| MYH6 | . | 2 |
| MYH9 | ALCL, TSG, fusion | 2 |
| NBPF15 | . | 2 |
| NOTCH3 | . | 2 |
| NRG3 | . | 3 |
| PAN3 | . | 2 |
| PDCD11 | . | 2 |
| PFKP | . | 3 |
| PHKA2 | . | 2 |
| PIK3C2G | . | 2 |
| PIK3R2 | . | 2 |
| PRIM2 | . | 2 |
| PRPF4B | . | 2 |
| PTDSS2 | . | 2 |
| PTPRF | . | 2 |
| RILPL1 | . | 2 |
| RNF213 | ALCL, fusion | 2 |
| RNF34 | . | 2 |
| RP1 | . | 2 |
| RPL6 | . | 3 |
| SCAF1 | . | 3 |
| SLC6A6 | . | 2 |
| SLTM | . | 2 |
| SMG1 | . | 2 |
| SNX13 | . | 2 |
| SREBF2 | . | 2 |
| SSH1 | . | 2 |
| SVEP1 | . | 2 |
| TARM1 | . | 2 |
| TBXA2R | . | 2 |
| TERT | melanoma, other tumor types, oncogene, TSG | 2 |
| TTN | . | 3 |
| UBR1 | . | 2 |
| UBR4 | . | 2 |
| UNC80 | . | 2 |
| VN1R4 | . | 2 |
| WDFY3 | . | 2 |
| WNT10B | . | 2 |
| WTIP | . | 2 |
| WWP2 | . | 2 |
| XK | . | 2 |
| ZNF429 | GBM | 3 |
| ZNF678 | . | 2 |
| ZNF845 | . | 2 |
| ZNF90 | . | 2 |

Note: CGC, cancer gene consensus; GBM, glioblastoma multiforme; TSG, tumor suppressor gene; HNSCC, head and neck squamous cell carcinoma; ALCL, anaplastic large cell lymphoma.

**Table S6. The arm-level CNVs of CCS.**

| Arm | # Genes | Amp frequency | Amp z-score | Amp q-value | Del frequency | Del z-score | Del q-value |
| --- | --- | --- | --- | --- | --- | --- | --- |
| 1p | 2121 | 0 | -1.15 | 0.924 | 0.1 | 0.551 | 0.702 |
| 1q | 1955 | 0.05 | -0.332 | 0.924 | 0.05 | -0.332 | 0.888 |
| 2p | 924 | 0.1 | 0.25 | 0.924 | 0.1 | 0.25 | 0.751 |
| 2q | 1556 | 0.1 | 0.47 | 0.924 | 0.1 | 0.47 | 0.727 |
| 3p | 1062 | 0.05 | -0.464 | 0.924 | 0.14 | 1.02 | 0.566 |
| 3q | 1139 | 0.05 | -0.502 | 0.924 | 0.1 | 0.245 | 0.751 |
| 4p | 489 | 0 | -1.43 | 0.924 | 0.1 | -0.0341 | 0.752 |
| 4q | 1049 | 0 | -1.34 | 0.924 | 0.1 | 0.143 | 0.752 |
| 5p | 270 | 0.14 | 0.623 | 0.924 | 0 | -1.43 | 0.923 |
| 5q | 1427 | 0.14 | 1.08 | 0.924 | 0 | -1.24 | 0.915 |
| 6p | 1173 | 0 | -1.29 | 0.924 | 0.14 | 0.971 | 0.566 |
| 6q | 839 | 0 | -1.26 | 0.924 | 0.24 | 2.35 | 0.076 |
| 7p | 641 | 0.33 | 3.74 | 0.00125 | 0 | -1.21 | 0.915 |
| 7q | 1277 | 0.29 | 3.4 | 0.00343 | 0 | -1.16 | 0.915 |
| 8p | 580 | 0.48 | 5.93 | 3.13E-08 | 0 | -1.08 | 0.915 |
| 8q | 859 | 0.71 | 9.98 | 0 | 0 | -0.771 | 0.888 |
| 9p | 422 | 0.05 | -0.614 | 0.924 | 0.14 | 0.768 | 0.605 |
| 9q | 1113 | 0.05 | -0.564 | 0.924 | 0.05 | -0.564 | 0.888 |
| 10p | 409 | 0 | -1.41 | 0.924 | 0.14 | 0.673 | 0.642 |
| 10q | 1268 | 0 | -1.34 | 0.924 | 0.05 | -0.577 | 0.888 |
| 11p | 862 | 0 | -1.34 | 0.924 | 0.14 | 0.843 | 0.593 |
| 11q | 1515 | 0 | -1.23 | 0.924 | 0.14 | 1.12 | 0.566 |
| 12p | 575 | 0 | -1.42 | 0.924 | 0.1 | -0.00821 | 0.752 |
| 12q | 1447 | 0 | -1.31 | 0.924 | 0.05 | -0.529 | 0.888 |
| 13q | 654 | 0.05 | -0.677 | 0.924 | 0.05 | -0.677 | 0.888 |
| 14q | 1341 | 0.1 | 0.316 | 0.924 | 0.05 | -0.45 | 0.888 |
| 15q | 1355 | 0 | -1.29 | 0.924 | 0.1 | 0.248 | 0.751 |
| 16p | 872 | 0.05 | -0.242 | 0.924 | 0.33 | 4.06 | 0.000809 |
| 16q | 702 | 0.05 | -0.281 | 0.924 | 0.33 | 3.95 | 0.000809 |
| 17p | 683 | 0.05 | -0.493 | 0.924 | 0.19 | 1.63 | 0.3 |
| 17q | 1592 | 0.05 | -0.382 | 0.924 | 0.1 | 0.408 | 0.737 |
| 18p | 143 | 0.1 | -0.0617 | 0.924 | 0.05 | -0.736 | 0.888 |
| 18q | 446 | 0.1 | 0.102 | 0.924 | 0.1 | 0.102 | 0.752 |
| 19p | 995 | 0 | -1.2 | 0.924 | 0.29 | 3.21 | 0.00673 |
| 19q | 1709 | 0 | -1.09 | 0.924 | 0.29 | 3.72 | 0.00134 |
| 20p | 355 | 0.05 | -0.567 | 0.924 | 0.19 | 1.49 | 0.35 |
| 20q | 753 | 0.1 | 0.196 | 0.924 | 0.1 | 0.196 | 0.752 |
| 21q | 509 | 0 | -1.43 | 0.924 | 0.1 | -0.0282 | 0.752 |
| 22q | 921 | 0.05 | -0.437 | 0.924 | 0.19 | 1.74 | 0.278 |
| Xp | 834 | 0 | -1.34 | 0.924 | 0.14 | 0.832 | 0.593 |
| Xq | 1312 | 0 | -1.26 | 0.924 | 0.14 | 1.03 | 0.566 |

Note: Amp, amplification; Del, deletion.

**Table S7. Arm-level CNV comparison among CCS, SKCM and TCGA Sarcoma.**

| Chromosome | CCS-ST | TCGA Sarcoma | SKCM |
| --- | --- | --- | --- |
| 7p,7q Amp | Y | - | Y |
| 8p,8q Amp | Y | - | Y |
| 16p Del | Y | Y | - |
| 16q Del | Y | Y | Y |
| 11p,11q Del | - | Y | Y |
| 19p,19q Del | Y | - | - |

Note: CCS-ST, clear cell sarcoma of soft tissue; CNV, copy number variations; SKCM, skin cutaneous melanoma; TCGA, the cancer genome atlas; Amp, amplification; Del, deletion; Y, yes (with arm-level CNV).

**Table S8. Focal amplifications of CCS.**

| chr_arm | wide peak boundarieswide | LengthofWidePeak | q value | residual q value | Number of genes in peak | Number of microRNAs in peak | genes in CGC |
| --- | --- | --- | --- | --- | --- | --- | --- |
| 10q21.2 | chr10:63525025-63847172 | 322148 | 0.012647 | 0.012647 | 2 | 0 | - |
| 13q14.11 | chr13:41533053-41665955 | 132903 | 0.067562 | 0.067562 | 2 | 0 | - |
| 14q11.2 | chr14:1-20299290 | 20299290 | 0.025736 | 0.025736 | 7 | 0 | - |
| 16p11.2 | chr16:28998950-29412502 | 413553 | 0.0032301 | 0.0032301 | 3 | 0 | - |
| 16q22.1 | chr16:69973298-70187269 | 213972 | 0.24435 | 0.24435 | 4 | 3 | - |
| 16q22.2 | chr16:70842098-71225312 | 383215 | 0.14836 | 0.14836 | 1 | 0 | - |
| 17q12 | chr17:36103873-36476420 | 372548 | 0.036181 | 0.078727 | 4 | 0 | - |
| 17q21.31 | chr17:43360129-44789284 | 1429156 | 0.0002387 | 0.0006915 | 17 | 3 | - |
| 17q23.3 | chr17:62080970-62527816 | 446847 | 0.017412 | 0.084834 | 9 | 2 | DDX5 |
| 1p11.2 | chr1:120523903-143424532 | 22900630 | 0.10703 | 0.10703 | 5 | 3 | NOTCH2 |
| 1p36.21 | chr1:12939097-13184130 | 245034 | 0.14836 | 0.14836 | 7 | 0 | - |
| 1q21.2 | chr1:148346286-149859759 | 1513474 | 0.0005469 | 0.0005469 | 23 | 0 | - |
| 1q43 | chr1:242681918-243390824 | 708907 | 0.040727 | 0.040727 | 2 | 0 | - |
| 21q22.11 | chr21:33697084-33834061 | 136978 | 0.19913 | 0.19913 | 3 | 0 | - |
| 5p15.33 | chr5:718098-825119 | 107022 | 0.0024884 | 0.0024884 | 1 | 0 | - |
| 5q35.3 | chr5:140874050-180915260 | 40041211 | 0.19913 | 0.19913 | 295 | 32 | CD74,CSF1R,EBF1,FGFR4,FLT4,ITK,NPM1,PDGFRB,ARHGAP26,TLX3,NSD1,PWWP2A |
| 6p22.1 | chr6:27439717-27890630 | 450914 | 0.0012011 | 0.0035816 | 18 | 0 | - |
| 6p22.2 | chr6:26017172-26371005 | 353834 | 0.0004129 | 0.000843 | 34 | 0 | HIST1H3B |
| 7q22.1 | chr7:102219573-102449147 | 229575 | 0.0012011 | 0.0012011 | 6 | 0 | - |
| 7q35 | chr7:143807305-144075748 | 268444 | 0.19913 | 0.19913 | 10 | 0 | - |

**Table S9. Focal deletions of CCS.**

| chr_arm | wide peak boundaries wide | Length of Wide Peak | q value | residual q value | Number of genes in peak | Number of microRNAs in peak | genes in CGC |
| --- | --- | --- | --- | --- | --- | --- | --- |
| 10q26.13 | chr10:124335633-124378138 | 42506 | 0.12518 | 0.12518 | 1 | 0 | - |
| 11q11 | chr11:51401066-55111583 | 3710518 | 0.20093 | 0.20093 | 4 | 0 | - |
| 14q11.2 | chr14:1-20445005 | 20445005 | 1.87E-08 | 1.87E-08 | 11 | 0 | - |
| 14q32.33 | chr14:107076001-107349540 | 273540 | 3.53E-09 | 0.0081075 |  | 0 | - |
| 17q21.2 | chr17:39253479-39432768 | 179290 | 0.044077 | 0.044077 | 16 | 0 | - |
| 19p12 | chr19:20726623-29702881 | 8976259 | 1.87E-08 | 1.87E-08 | 27 | 0 | ZNF429 |
| 19q13.31 | chr19:43242570-43569082 | 326513 | 2.90E-12 | 2.90E-12 | 8 | 0 | - |
| 19q13.33 | chr19:49520150-49560110 | 39961 | 0.0055973 | 0.0055973 | 9 | 0 | - |
| 19q13.42 | chr19:55246419-55331761 | 85343 | 6.38E-06 | 6.38E-06 | 5 | 0 | - |
| 1p36.13 | chr1:16900686-16920125 | 19440 | 8.75E-12 | 8.75E-12 | 1 | 0 | - |
| 1p36.21 | chr1:12882986-12955187 | 72202 | 5.49E-22 | 5.49E-22 | 5 | 0 | - |
| 1q21.1 | chr1:144619665-144822386 | 202722 | 0.0091949 | 0.0091949 | 2 | 0 | - |
| 1q21.2 | chr1:148009023-148345045 | 336023 | 0.007339 | 0.007339 | 3 | 0 | - |
| 1q44 | chr1:248096776-248732032 | 635257 | 0.020427 | 0.020427 | 23 | 0 | - |
| 2p11.2 | chr2:89339425-90153894 | 814470 | 4.69E-13 | 4.69E-13 |  | 0 | - |
| 2q11.2 | chr2:97852596-98175862 | 323267 | 1.17E-06 | 1.17E-06 | 2 | 0 | - |
| 9p21.3 | chr9:21140673-21481989 | 341317 | 0.0022445 | 0.0022445 | 17 | 1 | - |
| Xp11.23 | chrX:47970728-48317385 | 346658 | 0.13791 | 0.13791 | 9 | 0 | SSX1, SSX4 |

**Table S10. Clinical information of the CCS in GENIE database (N=35).**

| PATIENT_ID | SAMPLE_ID | AGE_AT_SEQ_REPORT | ONCOTREE_CODE | SAMPLE_TYPE | SEQ_ASSAY_ID | CANCER_TYPE | CANCER_TYPE_DETAILED |
| --- | --- | --- | --- | --- | --- | --- | --- |
| GENIE-VICC-179170 | GENIE-VICC-179170-unk-1 | 28 | CCS | Metastasis | VICC-01-T5A | Soft Tissue Sarcoma | Clear Cell Sarcoma |
| GENIE-MSK-P-0000849 | GENIE-MSK-P-0000849-T01-IM3 | 52 | CCS | Metastasis | MSK-IMPACT341 | Soft Tissue Sarcoma | Clear Cell Sarcoma |
| GENIE-MSK-P-0005989 | GENIE-MSK-P-0005989-T01-IM5 | 35 | CCS | Primary | MSK-IMPACT410 | Soft Tissue Sarcoma | Clear Cell Sarcoma |
| GENIE-GRCC-fb1fa651 | GENIE-GRCC-fb1fa651-metastasis-a | 30 | CCS | Metastasis | GRCC-CHP2 | Soft Tissue Sarcoma | Clear Cell Sarcoma |
| GENIE-UHN-573881 | GENIE-UHN-573881-ARC1 | 18 | CCS | Primary | UHN-48-V1 | Soft Tissue Sarcoma | Clear Cell Sarcoma |
| GENIE-UHN-838699 | GENIE-UHN-838699-ARC1 | 83 | CCS | Primary | UHN-48-V1 | Soft Tissue Sarcoma | Clear Cell Sarcoma |
| GENIE-DFCI-009363 | GENIE-DFCI-009363-7142 | 59 | CCS | Primary | DFCI-ONCOPANEL-2 | Soft Tissue Sarcoma | Clear Cell Sarcoma |
| GENIE-DFCI-006413 | GENIE-DFCI-006413-3436 | 19 | CCS | Metastasis | DFCI-ONCOPANEL-2 | Soft Tissue Sarcoma | Clear Cell Sarcoma |
| GENIE-MSK-P-0011423 | GENIE-MSK-P-0011423-T01-IM5 | <18 | CCS | Metastasis | MSK-IMPACT410 | Soft Tissue Sarcoma | Clear Cell Sarcoma |
| GENIE-MSK-P-0012694 | GENIE-MSK-P-0012694-T01-IM5 | 44 | CCS | Metastasis | MSK-IMPACT410 | Soft Tissue Sarcoma | Clear Cell Sarcoma |
| GENIE-JHU-01441 | GENIE-JHU-01441-01815 | 59 | CCS | Primary | JHU-50GP | Soft Tissue Sarcoma | Clear Cell Sarcoma |
| GENIE-DFCI-078015 | GENIE-DFCI-078015-298794 | 21 | CCS | Metastasis | DFCI-ONCOPANEL-3 | Soft Tissue Sarcoma | Clear Cell Sarcoma |
| GENIE-VICC-418549 | GENIE-VICC-418549-unk-1 | 46 | CCS | Primary | VICC-01-T7 | Soft Tissue Sarcoma | Clear Cell Sarcoma |
| GENIE-MSK-P-0021629 | GENIE-MSK-P-0021629-T01-IM6 | 54 | CCS | Metastasis | MSK-IMPACT468 | Soft Tissue Sarcoma | Clear Cell Sarcoma |
| GENIE-JHU-01930 | GENIE-JHU-01930-02352 | 55 | CCS | Primary | JHU-50GP | Soft Tissue Sarcoma | Clear Cell Sarcoma |
| GENIE-VHIO-034 | GENIE-VHIO-034-001 | 74 | CCS | Metastasis | VHIO-GENERAL-V01 | Soft Tissue Sarcoma | Clear Cell Sarcoma |
| GENIE-VICC-278704 | GENIE-VICC-278704-unk-1 | 52 | CCS | Metastasis | VICC-01-D2 | Soft Tissue Sarcoma | Clear Cell Sarcoma |
| GENIE-MSK-P-0027614 | GENIE-MSK-P-0027614-T02-IM6 | 19 | CCS | Metastasis | MSK-IMPACT468 | Soft Tissue Sarcoma | Clear Cell Sarcoma |
| GENIE-MSK-P-0028013 | GENIE-MSK-P-0028013-T01-IM6 | 40 | CCS | Primary | MSK-IMPACT468 | Soft Tissue Sarcoma | Clear Cell Sarcoma |
| GENIE-MSK-P-0029754 | GENIE-MSK-P-0029754-T01-IM6 | 65 | CCS | Primary | MSK-IMPACT468 | Soft Tissue Sarcoma | Clear Cell Sarcoma |
| GENIE-COLU-00202 | GENIE-COLU-00202-01 | 19 | CCS | Primary | COLU-CCCP-V1 | Soft Tissue Sarcoma | Clear Cell Sarcoma |
| GENIE-DFCI-141449 | GENIE-DFCI-141449-1301693 | 32 | CCS | Not Applicable or Heme | DFCI-ONCOPANEL-3 | Soft Tissue Sarcoma | Clear Cell Sarcoma |
| GENIE-MSK-P-0031637 | GENIE-MSK-P-0031637-T01-IM6 | 55 | CCS | Metastasis | MSK-IMPACT468 | Soft Tissue Sarcoma | Clear Cell Sarcoma |
| GENIE-MSK-P-0032329 | GENIE-MSK-P-0032329-T02-IM6 | 20 | CCS | Primary | MSK-IMPACT468 | Soft Tissue Sarcoma | Clear Cell Sarcoma |
| GENIE-MSK-P-0032409 | GENIE-MSK-P-0032409-T02-IM6 | 58 | CCS | Metastasis | MSK-IMPACT468 | Soft Tissue Sarcoma | Clear Cell Sarcoma |
| GENIE-MSK-P-0033510 | GENIE-MSK-P-0033510-T01-IM6 | 28 | CCS | Primary | MSK-IMPACT468 | Soft Tissue Sarcoma | Clear Cell Sarcoma |
| GENIE-MSK-P-0039378 | GENIE-MSK-P-0039378-T02-IM6 | 36 | CCS | Primary | MSK-IMPACT468 | Soft Tissue Sarcoma | Clear Cell Sarcoma |
| GENIE-MSK-P-0043602 | GENIE-MSK-P-0043602-T02-IM6 | 32 | CCS | Metastasis | MSK-IMPACT468 | Soft Tissue Sarcoma | Clear Cell Sarcoma |
| GENIE-MSK-P-0044247 | GENIE-MSK-P-0044247-T01-IM6 | 19 | CCS | Primary | MSK-IMPACT468 | Soft Tissue Sarcoma | Clear Cell Sarcoma |
| GENIE-UHN-OCT494304 | GENIE-UHN-OCT494304-ARC1 | 51 | CCS | Primary | UHN-OCA-V3 | Soft Tissue Sarcoma | Clear Cell Sarcoma |
| GENIE-MSK-P-0047115 | GENIE-MSK-P-0047115-T02-IM6 | 28 | CCS | Primary | MSK-IMPACT468 | Soft Tissue Sarcoma | Clear Cell Sarcoma |
| GENIE-MSK-P-0049830 | GENIE-MSK-P-0049830-T02-IM6 | 21 | CCS | Primary | MSK-IMPACT468 | Soft Tissue Sarcoma | Clear Cell Sarcoma |
| GENIE-UCSF-3724 | GENIE-UCSF-3724-7743T | 63 | CCS | Not Collected | UCSF-NIMV4-TO | Soft Tissue Sarcoma | Clear Cell Sarcoma |
| GENIE-DFCI-201132 | GENIE-DFCI-201132-2774592 | <18 | CCS | Primary | DFCI-ONCOPANEL-3.1 | Soft Tissue Sarcoma | Clear Cell Sarcoma |
| GENIE-JHU-04963 | GENIE-JHU-04963-05775 | 69 | CCS | Primary | JHU-50GP | Soft Tissue Sarcoma | Clear Cell Sarcoma |

**Material and Methods**

**Patients and samples**

The study retrospectively collected formalin-fixed, paraffin-embedded (FFPE) tumor samples and matched PBMC samples for genomic characterization. A total of 21 CCS samples (19 have paired normal samples) from Sun Yat-sen University Cancer Center were included. Twelve (57%) were women. Most patients were less than 50 years old and the median age at diagnosis was 32 years (20-58 years). Most patients (86%) were diagnosed at an early stage (stage I, 16 patients, stage II, 2 patients). Most of the tumors occurred at the foot, followed by the joints. The median relapse-free survival (RFS) time was 8 months. Ten patients died before the final follow-up (March 1, 2021). All samples were from patients treated at the Sun Yat-Sen University Cancer Center (SYSUCC) from April 2003 to April 2020 (Table S1). Each case was independently re-confirmed by a pathologist to have features of CCS using H&E staining, IHC examination (S100, HMB45, and Melan-A) and molecular characteristics (EWSR1-ATF1, EWSR1-CREB1 or EWSR1 break-apart FISH test positive). The study protocol was approved by the Institutional Review Board of SYSUCC. All patients provided written informed consent.

**Whole exome sequencing**The WES library construction and sequencing methods were consistent with those previously described^1^. The median paired-end 100 bp sequencing data of all CCS samples was 48.9 Giga base pair (Table S2), and after data preprocessing and deduplication, the median sequencing depths for tumors and normal samples were 231X and 188X, respectively. The hg19 genome mapping rate and other quality control characteristics of each sample are shown in Table S2.

**Identification of mutations**The bioinformatics analysis pipeline is shown in Fig. S1. The raw reads of each sample were filtered using fastp software^2^ and mapped to the human hg19 reference genome with MegaBOLT software. Then, the base quality score was recalibrated with GATK ^3,4^. The germline mutations were identified with normal samples using ‘HaplotypeCaller’ function and filtered by ‘VariantFiltration’ in GATK. The filtering parameters are as follows: -- filterExpression “QD < 2.0 | | MQ < 40.0 | | ReadPosRankSum < 8.0 | | FS > 60.0”. Multiple software programs were used for the detection of somatic SNVs and indels with paired tumor-normal samples, and at least one or more software-detected mutations were required before use in downstream analyses. Briefly, SNVs were detected using MuTect^5^, Mutect2^6^, Muse^7^, Strelka2^8^, and VarScan2^9^. Indels were detected using Strelka2^8^, Mutect2^6^ and VarScan2^9^. The mutations were then annotated with ANNOVAR^10^, and the false-positive sites were removed as much as possible through internal script filtering (Fig. S1). In brief, to remove false positives in the FFPE sample degradation and experimental processes, the following filtrations were used: (1) ‘FilterByOrientationBias’ in GATK was used to filter potential G/T and C/T artefacts caused by sample oxidation (8-oxoguanine) or the spontaneous deamination of cytosines; (2) The number of mutant reads was greater than 3, and the mutation frequency was greater than 5%; (3) The frequency in known databases (the Exome Aggregation Consortium, 1000Genomes) did not exceed 0.5% (0.1% for the two tumor only samples); (4) The reads supporting a mutation at the end (5 bp) were not considered; and (5) The mutation was not in the repetitive region of the genome (annotated by ANNOVAR^10^). Finally, a ‘panel of normal (PON)’ constructed by the PBMC samples was used to filter the germline and sequencing artefacts. For the two tumor samples without matched PBMC controls, mutations were detected in tumor-only mode, and ‘PON’ was used to remove germline mutations. The final mutation results are listed in Supplementary Table S3.

**Mutational signature and driver gene analysis**The SNVs obtained above were used to detect mutation signatures by MuSiCa^11^ and the ‘extractSignatures’ function in maftools^12^ simultaneously. The stable number of signatures was identified with ‘estimateSignatures’ of maftools^12^ based on the NMF method. Specifically, in the cophenetic matrix diagram, n value before Y-axis value drops sharply is signature value. In this study it appears to be at n = 2 (Figure S5). The gene signatures were further confirmed by MuSiCa^11^. Driver mutations were detected by OncodriveCLUSTL^13^, and mutations of cancer genes were analyzed by combining CCS mutation data in GENIE database to detect potential driver genes. The rs1242535815 mutation in the promoter region (5'flank) of *TERT* was confirmed by Sanger sequencing and screened in the GENIE database (version 9.0)^14^.

**Copy number analysis**Somatic CNVs were detected by the CNV workflow in GATK4. Normal samples were used as controls, and a PON was constructed to remove normal contamination as much as possible. The purity and ploidy of the tumor sample was evaluated by FACETs^15^ and used to correct any signal attenuation in the segment files according to the In Silico Admixture Removal (ISAR) method^16^. The sample with tumor purity lower than 20% was excluded from the following analysis. Then, the copy number segment file was used as the input of GISTIC2^17^ to detect broad and focal copy number variations. To obtain accurate somatic CNV results as much as possible, we used a noise threshold of 0.3, a broad length cut-off of 0.98 chromosome arms, a threshold q-value of 0.25, and a confidence level of 99%. The thresholds for gene copy number alterations were: gains: GISTIC score = 1; amplifications: GISTIC score = 2; deletions: GISTIC score = -2; losses: GISTIC score = -1. The amplifications of CDK4 and MDM2 were confirmed with fluorescence in situ hybridization (FISH). The fluorescent probes were from Anbiping (CDK4 probe cat#: F.01273-01, MDM2 probe cat#: F.01017-01).

**Pathway enrichment analysis**A total of 1875 genes in focal CNV regions were used for gene set enrichment analysis (GSEA). GSEA pathway enrichment analysis was based on the MSigDB v7.0 database, “CP:KEGG: KEGG gene sets” and “CP:REACTOME: Reactome gene sets” were used for GSEA analysis and contained 40312 genes used as background (http://www.gsea-msigdb.org/gsea/msigdb/annotate.jsp). The significant enrichment pathway was determined by hypergeometric testing (q <0.05).

**Comparison analysis**We downloaded the mutation data of SARC, SKCM and CCS from the cBioPortal (TCGA, http://www.cbioportal.org) and the GENIE database (https://www.synapse.org, version 9.0)^14^. The comparison of SNVs and the mutation load was performed with maftools package (v2.6.05)^12^ in R studio (R version 4.0.2), and the comparison of CNVs was performed based on the GISTIC results and visualized with IGV software (version 2.4.3). In GENIE9.0 database, somatic mutations were found in 26 CCS samples and somatic CNVs were found in 20 samples. Because CCS in GENIE database was from hundreds of genes with Panel sequencing, and the samples lacked clinical prognostic information, it was only used as a validation cohort for mutation of cancer-related genes (Fig. 1C) and pathway analysis (Fig. S9).

**Cell culture**
The U2OS osteosarcoma cell line and SU-CCS-1 clear cell sarcoma cell line were purchased from ATCC. All the cell lines were cultured in RPMI 1640 medium supplemented with 10% fetal bovine serum.

**Whole genome sequencing (WGS) and analysis of SU-CCS-1 cell line**The WGS library construction and sequencing methods were consistent with those previously described^18^. The library were sequenced on DNB-seq (BGI) platform and a total of 1,265,934,900 pair-end reads (100bp) were generated. MegaBOLT software was used for mapping and mutation calling of the WGS data. The mutations (SNV and indels) were annotated with ANNOVAR, and mutations with frequency more than 0.1% in the known databases (the Exome Aggregation Consortium, 1000Genomes) were excluded. CNVkit was used to detect and visualization CNVs in the cell line with default parameters. analysis^19,20^.

**RNA sequencing (RNA-seq) and analysis of CCS samples**RNA-seq libraries were constructed using the MGIEasy Library Prep Kit (1000005953, MGI) per manufacturer's recommendations. After library quality and quantity assessment, barcoded libraries were pooled and sequenced by an DNB-seq instrument (single-end 100 cycle reactions). RNA-seq was performed on 9 tumor FFPE tumor samples from CCS, and 6 samples were successfully sequenced. We quantified gene expression in these 6 samples by Kallisto^21^. The expression data of TCGA sarcoma (TCGA-SARC) were downloaded from the UCSC Xena database (https://xenabrowser.net) for comparison. In addition, CIBERSORT^22^ was used to estimate the proportion of immune cells in samples, and it was found that macrophages (P8, P9, P10) and CD4^+^ cells (P4, P14, P15) accounted for a high proportion in CCS, while CD8+ and NK cells accounted for a low proportion.

**Cell viability assays**
For the dose response curve, 100uL of SU-CCS-1 cells were seeded into 96-well plates (5000 cells/well) and individual wells were treated with increasing concentrations (0.01-100uM) of the respective drugs the following day. To compare the sensitivity of U2OS and SU-CCS-1 to PARP1 inhibitor and ATR inhibitor, both cell lines were treated with either 1uM olaparib, which is a concentration frequently used in studying the biological function of PARP1 inhibition in many different cancer cell lines, or 1uM AZD6738, which is a concentration close to IC50 in U2OS cells. Cells were cultured with drugs for 3 days in incubator, and cell viability was assessed by adding CellTiter-Glo reagent (Promega) and measuring luminescence using a plate reader (BioTek).

**Cell proliferation assays**
To determine the two drugs’ ability in sensitizing SU-CCS-1 cells to radiation, SU-CCS-1 cells were seeded on 6 cm dishes at optimized confluence (1.5×10^5^) in triplicate. The next day, the cells were treated with DDR inhibitors at their corresponding IC50 concentration (2uM olaparib or 0.5uM AZD6738), and 2 hours later, the cells were irradiated. The cells were then allowed to grow for 5 days. After the experiment, the trypan blue exclusion method was used to count the number of living cells.

**Western blotting**
The standard western blotting procedure were performed. In brief, cells were lysed and sonicated on ice in RIPA lysis buffer (R0278, Sigma) supplemented with Protease Inhibitor Cocktail (P8340, Sigma), Phenylmethylsulfonyl fluoride (PMSF; 10837091001, Sigma). 40 µg of protein were resolved on 6-12% SDS-polyacrylamide gels and transferred to PVDF membranes. The targeted proteins on the PVDF membranes were recognized by incubating with primary antibodies at 4°C overnight. After washing, the membranes were incubated with corresponding HRP conjugated secondary antibodies at room temperature for 1 hour, and the results were visualized using Gel-Doc system (Bio-Rad)^23^**.** Primary antibodies used in this study were: ATM (Abcam, cat#: ab199726, 1:1000 dilution), Chk2 (Santa Cruz Biotechnology, cat#: sc-5278, 1:1000 dilution), and alpha-Tubulin (Cell Signaling Technology, cat#, 3873, 1:1000 dilution).

**Immunohistochemistry (IHC)**The tumor tissue blocks were cut in 4-μm sections, and then dried at 65 °C for 1 h. The paraffin-embedded sections were deparaffinized with decreasing concentrations absolute alcohol. For antigen retrieval, tissue sections were boiled in Tris EDTA buffer (pH 9.0) at 100°C for 2.5 minutes in a pressure tank, and then cooled for 30 minutes at room temperature. The sections were washed with distilled water for 1 min, and incubated with primary antibodies PD-L1 (Cell Signalling, cat#:13684, dilution ratio 1:100) for 1 hour at 37°C. After being rinsed with PBS buffer, sections were treated with 3% H_2_O_2_ to eliminate endogenous peroxidase and rinsed again with PBS buffer. Sections were incubated with the secondary antibody (Zhongshan Jinqiao PV6000 Detection Kit) and incubated for 30 min at 37°C. At last, the sections were stained with DAB Peroxidase Substrate Kit (Zhongshan Jinqiao). PD-L1 expression was assessed by the percentage of PD-L1-expressing tumor cells (negative: <1% of tumor cells expressing PD-L1; positive: ≥1% of tumor cells expressing PD-L1). All original H&E- and IHC-stained slides were reviewed by two experienced pathologists. Images were taken with Olympus BX53 microscope with a DP80 CCD camera (Olympus).

**Fluorescence in situ hybridization (FISH)**The paraffin-embedded sections were deparaffinized with decreasing concentrations absolute alcohol. After being washed with distilled water, slides were heated in a water-isolation heating mode at 99°C for 20~30 minutes. Sections were digested with pepsin working solution (4mg/ml pepsin, 0.02M HCl) for 3~10 minutes and rinsed with SSC buffer at room temperature for 10 minutes. Then sections were dehydrated with gradient ethanol and air dried. Sections were incubated with probe mixture and placed on the in-situ hybridization instrument. The "Denat&Hyb" program was set to denature at 85°C for 5 minutes, and hybridize at 37°C for 8 hours. After rinsing with SSC buffer, sections were further stained with DAPI/anti-fade buffer and viewed on fluorescent microscope for signal. The fluorescent probes were from Anbiping (CDK4 probe cat#: F.01273-01, MDM2 probe cat#: F.01017-01). Images were taken with Olympus BX51 fluorescent microscope with a DP80 CCD camera (Olympus).

**Statistical analysis**Fisher’s exact test was used for discrete variables, the survival probability was calculated, and the difference in survival was analysed using the log-rank test. For mutation visualization, we use maftools (v2.6.05), ggplot2 (v3.3.3) and ggsignif (v0.6.1); for survival analysis, we use survival (v3.1-12), survminer (v0.4.9). All statistical analyses were performed in R studio (R 4.0.2). If not specified, statistical significance was considered if P value < 0.05.

References

1. Chen, C. *et al.* Molecular Profiles and Metastasis Markers in Chinese Patients with Gastric Carcinoma. *Scientific Reports* **9**(2019).

2. Chen, S., Zhou, Y., Chen, Y. & Gu, J. fastp: an ultra-fast all-in-one FASTQ preprocessor. *Bioinformatics* **34**, i884–i890 (2018).

3. DePristo, M.A. *et al.* A framework for variation discovery and genotyping using next-generation DNA sequencing data. *Nat Genet* **43**, 491-8 (2011).

4. McKenna, A. *et al.* The Genome Analysis Toolkit: a MapReduce framework for analyzing next-generation DNA sequencing data. *Genome Res* **20**, 1297-303 (2010).

5. Cibulskis, K. *et al.* Sensitive detection of somatic point mutations in impure and heterogeneous cancer samples. *Nat Biotechnol* **31**, 213-9 (2013).

6. McKenna, A. *et al.* The Genome Analysis Toolkit: a MapReduce framework for analyzing next-generation DNA sequencing data. *Genome Res* **20**, 1297-303 (2010).

7. Fan, Y. *et al.* MuSE: accounting for tumor heterogeneity using a sample-specific error model improves sensitivity and specificity in mutation calling from sequencing data. *Genome Biol* **17**(2016).

8. Kim, S. *et al.* Strelka2: fast and accurate calling of germline and somatic variants. *Nature Methods* **15**, 591-594 (2018).

9. Koboldt, D.C. *et al.* VarScan 2: somatic mutation and copy number alteration discovery in cancer by exome sequencing. *Genome Res* **22**, 568-76 (2012).

10. Wang, K., Li M & Hakonarson H. ANNOVAR: functional annotation of genetic variants from high-throughput sequencing data. *Nucleic Acids Res* **38**, e164 (2010).

11. Díaz-Gay, M., Vila-Casadesús, M. & Franch-Expósito, S. Mutational Signatures in Cancer (MuSiCa): a web application to implement mutational signatures analysis in cancer samples. *BMC Bioinformatics* **19**, 224-229 (2018).

12. Mayakonda, A., Lin, D.-C., Assenov, Y., Plass, C. & Koeffler, H.P. Maftools: efficient and comprehensive analysis of somatic variants in cancer. *Genome Res* **28**, 1747-1756 (2018).

13. Arnedo-Pac, C., Mularoni, L., Muinos, F., Gonzalez-Perez, A. & Lopez-Bigas, N. OncodriveCLUSTL: a sequence-based clustering method to identify cancer drivers. *Bioinformatics* **35**, 5396 (2019).

14. AACR Project GENIE: Powering Precision Medicine through an International Consortium. *Cancer Discov* **7**, 818-831 (2017).

15. Shen, R. & Seshan, V.E. FACETS: allele-specific copy number and clonal heterogeneity analysis tool for high-throughput DNA sequencing. *Nucleic Acids Research* **44**, e131-e131 (2016).

16. Zack, T.I. *et al.* Pan-cancer patterns of somatic copy number alteration. *Nat Genet* **45**, 1134-40 (2013).

17. Mermel, C.H. *et al.* GISTIC2.0 facilitates sensitive and confident localization of the targets of focal somatic copy-number alteration in human cancers. *Genome Biol* **12**, R41 (2011).

18. Li, L. *et al.* The ChinaMAP reference panel for the accurate genotype imputation in Chinese populations. *Cell Research* (2021).

19. Sun, Y. *et al.* Single-cell landscape of the ecosystem in early-relapse hepatocellular carcinoma. *Cell* **184**, 404-421 e16 (2021).

20. Talevich, E., Shain, A.H., Botton, T. & Bastian, B.C. CNVkit: Genome-Wide Copy Number Detection and Visualization from Targeted DNA Sequencing. *PLoS Comput Biol* **12**, e1004873 (2016).

21. Bray, N.L., Pimentel, H., Melsted, P.A.-O. & Pachter, L. Near-optimal probabilistic RNA-seq quantification. *Nature Biotechnology* **34**, 525–527 (2016).

22. Newman, A.M. *et al.* Robust enumeration of cell subsets from tissue expression profiles. *Nature Methods* **12**, 453-457 (2015).

23. Taparra, K. *et al.* O-GlcNAcylation is required for mutant KRAS-induced lung tumorigenesis. *J Clin Invest* **128**, 4924-4937 (2018).
